# Scalable Matrigel‐Free Suspension Culture for Generating High‐Quality Human Liver Ductal Organoids

**DOI:** 10.1111/cpr.70033

**Published:** 2025-04-01

**Authors:** Senyi Gong, Kangxin He, Yu Liu, Xingyu Luo, Kamran Ashraf, Jinzhao He, Weifeng Li, Lihua Yang, Touseef Ur Rehman, Mingwei Shen, Qinbiao Yan, Ali Mohsin, Shusen Zheng, Zhe Yang, Meijin Guo

**Affiliations:** ^1^ State Key Laboratory of Bioreactor Engineering East China University of Science and Technology Shanghai China; ^2^ Department of Hepatobiliary and Pancreatic Surgery, Key Laboratory of Artificial Organs and Computational Medicine in Zhejiang Province, Shulan (Hangzhou) Hospital, Shulan International Medical College Zhejiang Shuren University Hangzhou China; ^3^ State Key Laboratory for Diagnosis and Treatment of Infectious Diseases, National Clinical Research Center for Infectious Diseases, National Medical Center for Infectious Diseases, Collaborative Innovation Center for Diagnosis and Treatment of Infectious Diseases, the First Affiliated Hospital Zhejiang University School of Medicine Hangzhou China

**Keywords:** large‐scale production, liver ductal organoids, liver transplantation, matrigel free suspension culture

## Abstract

Liver transplantation is currently the sole definitive treatment option for end‐stage liver failure. However, a significant shortage of donors prevails due to high clinical demands. Recently, human liver organoids have shown significant potential in regenerative medicine for liver diseases. Nevertheless, current static cultures of organoids grown in well‐plates heavily rely on extracellular matrix hydrogels (Matrigel), thereby limiting both the scalability and quantity of organoid culture. In this study, we present a groundbreaking culture mode that eliminates all reliance on extracellular matrix hydrogels, enabling the successful preparation of functional human liver ductal organoids (LDOs) based on the cell suspension culture mode in a mechanically stirred bioreactor. Initially, the developed suspension culture in a 6‐well plate without matrigel was proven to support robust growth of liver ductal organoids with an average size 2.6 times larger than those obtained in static culture, and with a high organoid survival rate exceeding 90%. Also, the transcriptome profile reveals that suspension culture activates the phosphatidylinositol 3‐kinase (PI3K) signalling pathway through mechanical signal transduction, thereby promoting hepatobiliary characteristics. Then, a controllable and scalable bioprocess for liver ductal organoid culture was developed and successfully scaled up to a 50 mL flask bioreactor with a working volume of 15 mL. Finally, animal experiments indicated that the transplantation of liver ductal organoids harvested from suspension culture can effectively alleviate liver injury and inflammation, demonstrating the feasibility of large‐scale production of liver ductal organoids cultivated in suspension culture with an improved extracellular matrix environment.

## Graphical abstract

A novel matrigel‐free suspension culture enhances the generation of high‐quality human liver ductal organoids, which shows remarkable efficacy in mice transplantation.

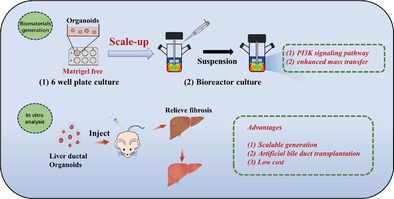

## 1 Introduction

Currently, liver transplantation is the only curative option for the treatment of end‐stage liver failure [1, 2]. However, the supply of donor livers is greatly insufficient to meet clinical needs [3, 4]. To overcome this challenge, various regenerative medicine methods have been explored, such as cell transplantation [5], bioartificial organs [6], and liver support devices [7]. Among these, cell transplantation is a promising alternative method for liver transplantation [8]. The implementation of cell therapy is hampered by the lack of scalable cell preparation of liver parenchymal cells due to the complexity of cultivation conditions and the propensity for cells to lose their liver characteristics [9, 10]. Organoids hold particular potential for tissue repair as they retain key functions and characteristics of their original tissue [11, 12, 13]. For clinical applications of regenerative tissue, several important considerations need to be addressed, including immune responses and the number of cells available [14, 15, 16]. Using autologous cell sources is a possible solution to avoid immune rejection [17, 18]. Therefore, adult stem cell‐derived organoids offer distinct advantages as a cell source for regenerative medicine compared to allograft‐derived or iPSC‐derived organoids [19, 20]. Currently, liver ductal organoids have been established from bile duct progenitor cells by a 3D culture using the matrigel embedding method, which allows for bipotential differentiation capacity into both hepatocyte and cholangiocyte lineages [21]. A proof‐of‐concept work reported that liver ductal organoids exert excellent regenerative potential in repairing the intrahepatic bile duct structure in both mouse and human liver [22].

At present, many factors limit the application of scaled organoid preparation in regenerative medicine purposes. Firstly, organoid culture pathways are typically carried out under static conditions, with matrix gel carriers facilitating cell differentiation and self‐assembly [23, 24]. Three‐dimensional cell culture enables the stimulation of organs, diseases, and human development [25, 26, 27]. The most used matrigel is derived from a mixture of proteins secreted by mouse sarcoma cells, providing structural support and extracellular signalling for cells [28, 29]. However, animal‐derived matrigel has a certain probability of containing residual animal protein components and may exhibit differences in matrix adhesion due to individual contrasts and the physiological status of animals [30, 31]. Secondly, considering the physical and chemical properties of the matrigel, researchers must employ more sophisticated experimental protocols to prevent premature gel solidification [24, 28]. Compared to suspension culture systems, the static expansion process requires a larger surface area per unit of growth and more manual labour [32, 33]. This introduces uncertainty into scientific research, affecting the repeatability of experiments and the reliability of results. Last but not least, mechanical forces have been demonstrated to play a significant role in the formation of life, with the continuous advancement of molecular biology techniques [34, 35, 36]. Liver organoids, with their complex cell growth microenvironments, present dynamic biochemical clues and mechanical stimuli that drive cell mechanical transduction through mechanical sensing, ultimately leading to changes in tissue structure [37, 38]. We speculate that traditional static culture cannot provide the necessary mechanical forces to promote cell growth, which may limit the rate and quality of cell growth [39, 40, 41]. Therefore, it is necessary to develop a novel cultivation strategy that meets the needs of regenerative medicine while addressing current cultivation limitations, removing the need for matrigel and providing the necessary mechanical forces to promote cell growth.

As liver ductal organoids can be generated from a small mass of a patient's liver without gene editing, engineered liver ductal organoids are considered a potential cell source for in vitro transplantation, reconstructed from autologous cells [42]. For instance, it is estimated that 10% of liver mass contains approximately billions of cells, which could be helpful for curing liver failure. However, culturing billions of cells requires a large amount of culture space and time in static culture, which hinders the research and development of engineered liver ductal organoids for liver injury repair. Current research teams have developed strategies for the suspension culture of liver ductal organoids, but these methods have not completely departed from static culture, as the process still requires the addition of matrigel, limiting further clinical applications [33, 43, 44, 45]. Therefore, it is necessary to develop new suspension culture methods to overcome the shortcomings of static culture. Firstly, suspension culture can expand the cultivation volume, greatly improving space limitations, and reducing labor time. Secondly, it can increase the number of cultured cells to meet clinical needs [46]. Thirdly, suspension culture can promote the expansion of liver ductal organoids.

**FIGURE 1:**
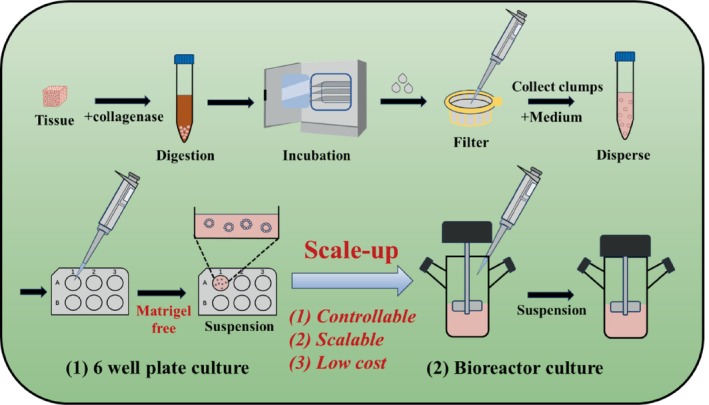
Scheme of the suspension culture mode of liver ductal organoids.

Here, we report a new suspension culture method for generating liver ductal (LDOs) organoids. In contrast to traditional static culture, this method does not require the use of matrigel, which greatly reduces production costs and improves experimental reliability. As illustrated in Figure 1, this is the first time that suspension culture of organoids has been achieved in a 6 well plate without Matrigel. We then extended the culture system to a flask bioreactor, successfully achieving the goal of ‘scale up’ for sufficient cell amount. The characteristics of the strategy developed in this work are as follows: (1) It is controllable during the culture process; (2) The culture system is scalable with high cell density; (3) The cost is lower due to the replacement of matrigel. More importantly, it was observed that the growth rate of organoids improved in this new culture system, resulting in a higher yield of cells harvested under the same conditions. Transcriptomic profiling revealed that suspension culture activates the PI3K signalling pathway through mechanotransduction, promoting hepatobiliary characteristics. Furthermore, transplantation effects of suspension cultured organoids were validated in vivo, indicating that the organoids obtained through scaled‐up preparation possess excellent liver repair capabilities, alleviating fibrosis and inflammation in mice with liver injury. Overall, this study provides an alternative suspension culture for large‐scale preparation of organoids in regenerative medicine.

## 2 Experiments

### 2.1 Preparation and Culture of Human Liver Organoids

Liver tissues were collected during liver procedures at Shulan (Hangzhou) Hospital, Hangzhou, China. The use of human tissue for research purposes was approved by the Medical Ethical Council of the Shulan (Hangzhou) Hospital (KY2024020). Firstly, liver specimens were mechanically minced with scissors into about 1 mm^3^ cubes, and the tissue fragments were washed with PBS for 3 times to remove impurities. Then, the tissue fragments were enzymatically digested with DMEM/F12 containing 0.1 mg/mL DNase I and 0.5 mg/mL collagenase type III for 30 min at 37°C. The cell clusters trapped on the 100 μm filters were collected and suspended in DMEM/F12 and centrifuged at 300 × g for 5 min. The precipitated cell clusters were resuspended in human culture medium and placed in one well of non‐treated 6‐well plates with 3 mL culture medium (HepatiCult Organoid Kit, Stemcell). The 6‐well plates were transferred to the orbital shaker in a 37°C incubator as soon as possible. The shaker was set at 90 r/min. After every 2 days, the 6‐well plate can be slanted to allow the organoids to settle. Subsequently, the medium can be carefully aspirated and replaced with fresh medium.

### 2.2 Detection of the Growth of Organoids

To detect the growth of organoids, microscopes were used to photograph the organoids on the third, seventh, and fourteenth days. The sizes of organoids under different culture methods were measured, and then the average sizes were calculated. To detect the viability of organoids, suspension cultured liver ductal organoids were collected and washed twice with PBS. Then the organoids were incubated with DMEM/F12 containing 10 mg/mL collagenase type III for 30 min at 37°C. The cell suspension from the digested organoids was centrifuged and collected for use. An automatic cell fluorescence analyser was used to determine the viability of organoids (Countstar Mira FL, Shanghai Ruiyu Biotech Co. Ltd., China).

### 2.3 Quantitative Real‐Time PCR

Before the quantitative real‐time PCR assay, liver ductal organoids in different conditions were collected and washed twice with PBS. Then the organoids were incubated with DMEM/F12 containing 1 mg/mL collagenase type III for 30 min at 37°C. The cell suspension from the digested organoids was centrifuged and collected for use. Total RNA was extracted from organoids and liver tissues using the cDNA synthesis kit and reverse transcribed into cDNA using qPCR master mix (Vazyme). Then the expressions of genes were detected by qPCR. The sequences of primers are shown in Table S1.

### 2.4 Flow Cytometry Assay

Before the flow cytometry assay, suspension cultured liver ductal organoids were collected and washed twice with PBS. Then the organoids were incubated with DMEM/F12 containing 10 mg/mL collagenase type III for 30 min at 37°C. The cell suspension from the digested organoids was centrifuged and collected for use.

### 2.5 Functional Validation of Liver Ductal Organoids

In order to verify the liver ductal function of organoids through the Rhodamine 123 efflux test, organoids and Rhodamine 123 (100 μM) were combined and incubated in HBSS for 10 min. The sample was subjected to three washes with HBSS, followed by imaging analysis. In order to inhibit the activity of the MDR1 transporter protein, the second group of organoids was mixed with a medium containing 20 μM verapamil for 30 min. Then change the culture medium to HBSS containing 100 μM Rhodamine 123 and 20 μM verapamil. After incubation for 5 min, the sample was washed three times with HBSS. Fluorescence images of organoids were obtained using Leica TCS SPE confocal microscopy. Fluorescence image quantification was measured using the MShot Image Analysis System V1.1.4.

### 2.6 FXR Signalling Pathway Activation Experiment

For the determination of bile acid homeostasis, organoids in both static and dynamic culture modes were pre‐treated with DMSO (Control) or 10 × 10^−6^ M GW4064 (FXR agonist; yeasen) for 48 h, followed by the assessment of bile acid transporter gene expression via qPCR.

### 2.7 RNA‐ Sequencing Analysis

Organoids derived from static and suspension cultures were collected and subjected to transcriptome sequencing and bioinformatics analysis by Applied Protein Technology (Shanghai, China).

### 2.8 Biliary Fibrosis Response Model Phenotype

Prior to the commencement of the experiment, organoids were treated with 25 ng/mL TGFβ (Sigma‐Aldrich) for 48 h. RNA was isolated, and the expression of biliary fibrosis genes was evaluated through qPCR.

### 2.9 Western Blot Analysis

To examine the effect of the suspension culture on the expression of relevant proteins. The suspension cultured organoids, static cultured organoids, and single cells digested from tissues were cultured under the same condition. The samples were then lysed with RIPA buffer containing 1 × PMSF and EDTA for 5 min at 4°C, and the lysates were collected with a cell scraper. To prevent incomplete cell lysis, the cells were further broken by an ultrasonic cell splicer, and the protein samples in the supernatant were obtained by centrifugation (4°C, 12000 rpm, 20 min). Protein concentration was quantified by the BCA Protein Assay kit. After separating the membrane proteins into bands of different molecular weights by electrophoretic separation, the proteins were transferred to a 0.22 μm polyvinylidene difluoride membrane and blocked with a 5% skim milk solution for 2 h. Prior to fluorescence imaging using the ECL kit, secondary antibodies for Hnf‐4α and Krt19 were incubated separately with PVDF membranes and followed by incubation of secondary antibodies overnight at 4°C.

### 2.10 Establishment of Mouse Models

Specific‐pathogen‐free (SPF) male 8‐week‐old NOD‐SCID‐γcnull (NSG) mice were purchased from Shanghai Model Organisms (China, Shanghai) and housed under controlled 12‐h light and dark cycles. Mice were randomly divided into two groups: the normal control group (Control) and the liver ductal cell transplantation group (Organoid), with five mice in each group. From day‐14 to day 0, both groups of mice were fed a 0.1% 3,3′‐diindolylmethane (DDC) diet. DDC can promote the accumulation of protoporphyrin, damage the biliary epithelium, and cause liver injury. Starting from day 0, mice were fed a control diet. The health status of the mice was monitored daily, and body weight was recorded every 2 days. On day 30, all mice were euthanized.

### 2.11 Liver Ductal Cell Transplantation

Before the transplantation experiment begins, organoids are first dissociated into single cells according to the previously described methods. The cells are washed with PBS and resuspended in 100 μL of PBS (10^6 cells). After the mouse is anaesthetised, the distal common bile duct is clamped with forceps, and the infusion is transferred to the liver, allowing biliary epithelial cells to enter the liver in a retrograde manner through the extrahepatic biliary tree.

### 2.12 Safety Evaluation of Cell Transplantation

To verify whether any human cells were transplanted into other organs of the mice, the liver ductal organoids were first dissociated into single cells and transplanted into the mice model as described above. After 14 days, the mice were sacrificed, and the heart, spleen, lung, and kidney were collected. The organs were dissociated into single cells using collagenase (1 mg/mL). The proportion of human liver ductal cells positive for KRT19 in the heart, spleen, lung, and kidney was analysed by flow cytometry. The positive control was human liver ductal cells cultured in vitro derived from the liver ductal organoids.

### 2.13 Serum Analysis

After confirming the death of the mice, blood is collected via the orbital sinus and centrifuged at 4°C at 6000 × g for 10 min. All sample analyses are performed on a Clinical Chemistry Analyser (cobas c311), using reagents and analytical protocols provided by Roche.

### 2.14 Histological Analysis

Tissues were fixed in 4% paraformaldehyde (Wako) for 24 h at 4°C or in 10% formalin overnight at room temperature and then embedded in paraffin. Paraffin‐embedded sections (4 μm) were dewaxed, rehydrated, and subjected to either haematoxylin and eosin (HE) staining or immunohistochemical staining. All antibodies were diluted with PBS with 5% donkey serum and 0.1% Triton X‐100. The sections were incubated with primary antibody overnight at 4°C, followed by incubation with secondary antibody for 1 h at room temperature. The stained sections were visualised using an Olympus BX50F4 microscope (Olympus Optical, Tokyo, Japan). The antibodies used for immunostaining are listed.

### 2.15 Statistical Analysis

Experiments were performed in triplicate and values are shown as means standard errors of the mean. Statistical analyses were performed using GraphPad Prism 9.0 for Windows (GraphPad Software, San Diego, CA, USA). Differences were judged as follows: * = *p* < 0.05, ** = *p* < 0.01, *** = *p* < 0.001. For microarray analyses, genes with an adjusted *p* < 0.05 were considered as differentially expressed in all comparisons unless mentioned otherwise.

## 3 Results

### 3.1 Growth of Liver Organoids Cultivated in Static and Suspension Cultures

**FIGURE 2:**
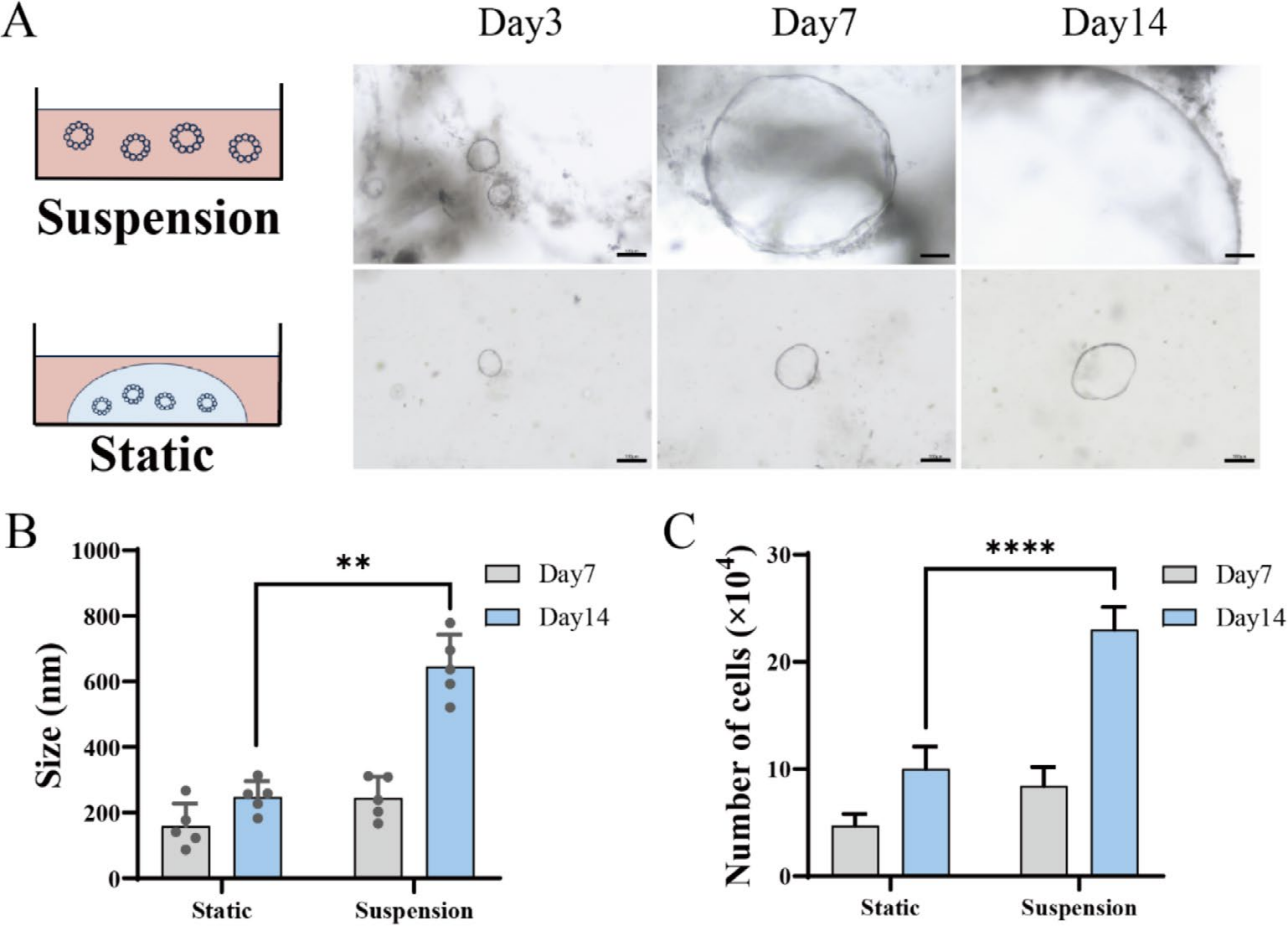
Size and cell number of liver organoids cultivated in 2D static and 3D suspension cultures. (A) Morphology of liver organoids. Scale bar: 100 μm. (B) size comparison of human liver ductal organoids grown under suspension and static culture conditions. (*n* = 5). (C) The cell number of liver organoids grown in static and suspension cultures.

To evaluate the growth advantages of the suspension cultured organoids, the size and cell number of liver organoids were compared under 2D static culture with matrigel and 3D suspension culture in a bioreactor without matrigel. As shown in Figure 2A, liver ductal organoids cultured in suspension developed cystic structures with cavities similar to those observed in static culture. However, the growth rate of the organoids in suspension culture was generally superior to that of static culture. The diameter of the suspension cultured organoids reached 800 μm on day 5 and 2000 μm on day 14, respectively. The size of the organoids in suspension culture was thrice that of those in static culture. Therefore, the developed suspension culture is suitable for liver ductal organoids growth, with advantages in facilitating organoid formation. The proposed suspension culture method demonstrated enhanced volumetric capacity. In the absence of an adhesive matrix, organoids with larger sizes could still be obtained, possibly due to the shear force and support provided by suspension culture through fluid dynamics, which stimulates the signalling pathways related to cell survival and mitosis [47]. In addition, measurements from both groups indicated that the average size of organoids cultured in suspension over 14 days was 2.6 times that of static culture (Figure 2B). Compared to the previous literature about suspension culture with matrigel [43, 44, 45], the strategy developed in this study offers an effective matrigel‐free suspension culture mode, eliminating the influence of the matrigel, reducing reagents and labour cost, and promoting a faster growth rate of organoids. The cell number of cultivated organoids obtained from suspension culture was greater than that from static culture on Day 7 and Day 14 under the same condition of initial cells and culture time (Figure 2C). This phenomenon could potentially be attributed to the transmission of mechanical forces, particularly shear stress, entering the interior of the cell via structures such as the cell membrane and cytoskeleton, thereby influencing various biological functions of the cell [48, 49]. During the processes of cell survival and mitosis, shear stress can modulate intracellular signalling pathways, thereby promoting cell proliferation and survival [34, 50].

### 3.2 Suspension Cultured Liver Ductal Organoids Exhibit Characteristics of Cholangiocytes

**FIGURE 3:**
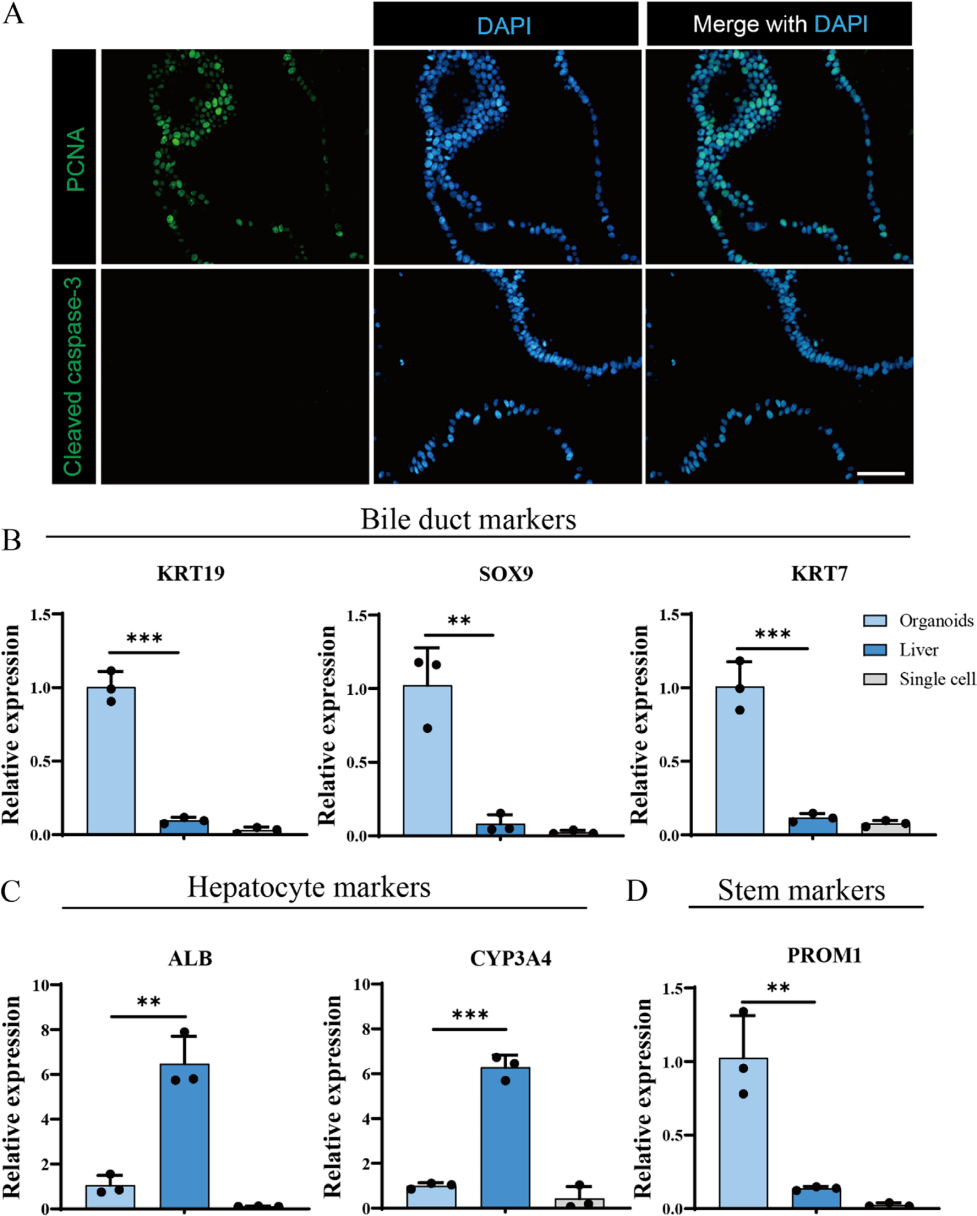
Characterisation of human liver ductal organoids. (A) Immunofluorescence images of suspension cultured liver ductal organoids stained for PCNA and Cleaved caspase‐3 markers. Scale bar: 100 μm. Relative gene expression of (B) liver ductal genes; (C) hepatocyte genes; (D) stemness genes biomarker of liver ductal organoids, primary hepatocytes, and primary hepatocytes cultured in suspension condition by qPCR detection (*n* = 3).

Liver ductal organoids typically possess bipotent differentiation potential into cholangiocytes and hepatocytes. Therefore, we investigated the differentiation characteristics of liver ductal organoids under suspension culture conditions. Firstly, the cell viability of organoids was characterised by evaluating immunofluorescence of proliferation and apoptotic biomarkers. As shown in Figure 3A, liver ductal organoids exhibited superior cell viability, as indicated by a positive PCNA staining. However, cleaved caspase‐3, which serves as a marker for cellular apoptosis, was not detected in the cells. This observation indicates that the cultured organoids had not experienced apoptosis. In this suspension culture system, organoids grew in clusters. We compared the levels of characteristic markers among three groups (Figure 3B–D): liver ductal organoids in suspension culture (Organoids), primary hepatocytes (Liver), and primary hepatocytes cultured for 14 days under the same culture conditions (Single cell). The expression of cholangiocyte‐specific markers KRT19, KRT7, and SOX9 in the suspension‐cultured liver ductal organoids was much higher compared to primary hepatocytes, respectively. In contrast, the expression of hepatocyte biomarkers, such as ALB and CYP3A4, was relatively lower in organoids compared to primary hepatocytes. These findings indicate that liver ductal organoids cultured under suspension conditions maintained excellent proliferative potential and bipotent stemness, exhibiting more characteristics of cholangiocytes. To validate the results, a control group was established by culturing primary hepatic tissue cells (as single cells) under the same conditions. The results showed that hepatocytes cultured in suspension as single cells, rather than in clusters, lost their stemness and hepatocyte characteristics in vitro.

### 3.3 Suspension Culture Enhances the Generation of High‐Quality Human Liver Ductal Organoids

**FIGURE 4:**
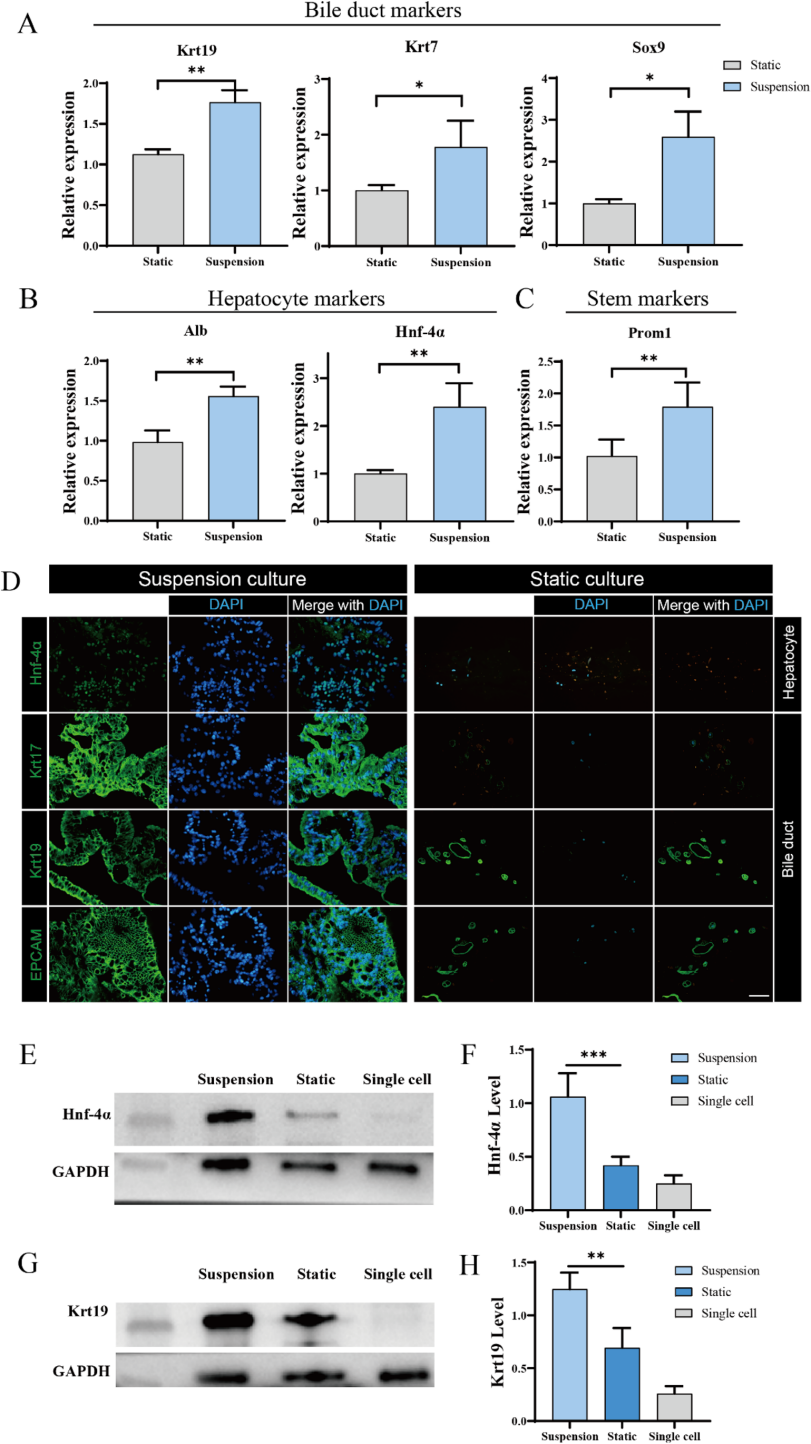
Comparison of organoid quality under static and suspension culture conditions. Relative gene expression of biomarker of organoids cultured under static and suspension conditions by qPCR detection: (A) liver ductal genes; (B) hepatocyte genes; (C) stemness genes. (D) Representative fluorescence microscopy images of organoids under suspension and static culture conditions, immune stained for cholangiocyte lineage markers (KRT19, KRT7, EPCAM) and hepatocyte lineage markers (HNF‐4a). Scale bar: 100 μm. (E) The images of western blot analysis and (F) expression level of Hnf‐4α protein in groups of suspension cultured organoids, static cultured organoids and single cells. (G) The images of western blot analysis and (H) expression level of Krt19 protein in groups of suspension cultured organoids, static cultured organoids and single cells.

After confirming the cholangiocyte characteristics of organoids cultured in suspension, we compared the differentiation levels of organoids cultured in suspension and static conditions. Liver ductal organoids were analysed by qPCR and immunofluorescence to determine the expression of markers specific to cholangiocytes and hepatocytes. The qPCR results demonstrated that organoids cultured in suspension exhibited enhanced lineage marker levels and elevated stemness (Figure 4A–C). In the immunofluorescence assays, liver ductal organoids exhibited cystic structures expressing cholangiocyte lineage markers, such as KRT19, KRT7, and EPCAM, whereas the hepatocyte lineage marker HNF‐4a was relatively low (Figure 4D). Compared with statically cultured organoids, those cultured in suspension exhibited more pronounced expression of cholangiocyte lineage markers and hepatocyte lineage markers. To further detect liver ductal‐related biomarkers, we conducted western blot experiments. Separate primary cells that had undergone the same suspension cultivation conditions were used for comparison. As shown in Figure 4E–H, the suspension‐cultured organoids exhibited stronger expression of liver and liver ductal lineage markers compared to traditional matrigel‐cultured organoids. According to the statistical chart, the expression of liver and liver ductal markers in suspension culture was almost twice that in static culture mode. However, individual cells lost the characteristics of the liver and liver ductal structures after being cultured in vitro. This indicates that the organoids can maintain the characteristics of liver ducts in vitro culture, and suspension‐cultured organoids are more conducive to preserving these characteristics. These findings indicate that liver ductal organoids cultured under suspension conditions, which grow from primary hepatocytes with bipotent stemness, exhibit enhanced hepatic ductal characteristics of higher quality compared to those cultured under static conditions.

### 3.4 Verification of Mature Bile Duct Cell Function

**FIGURE 5:**
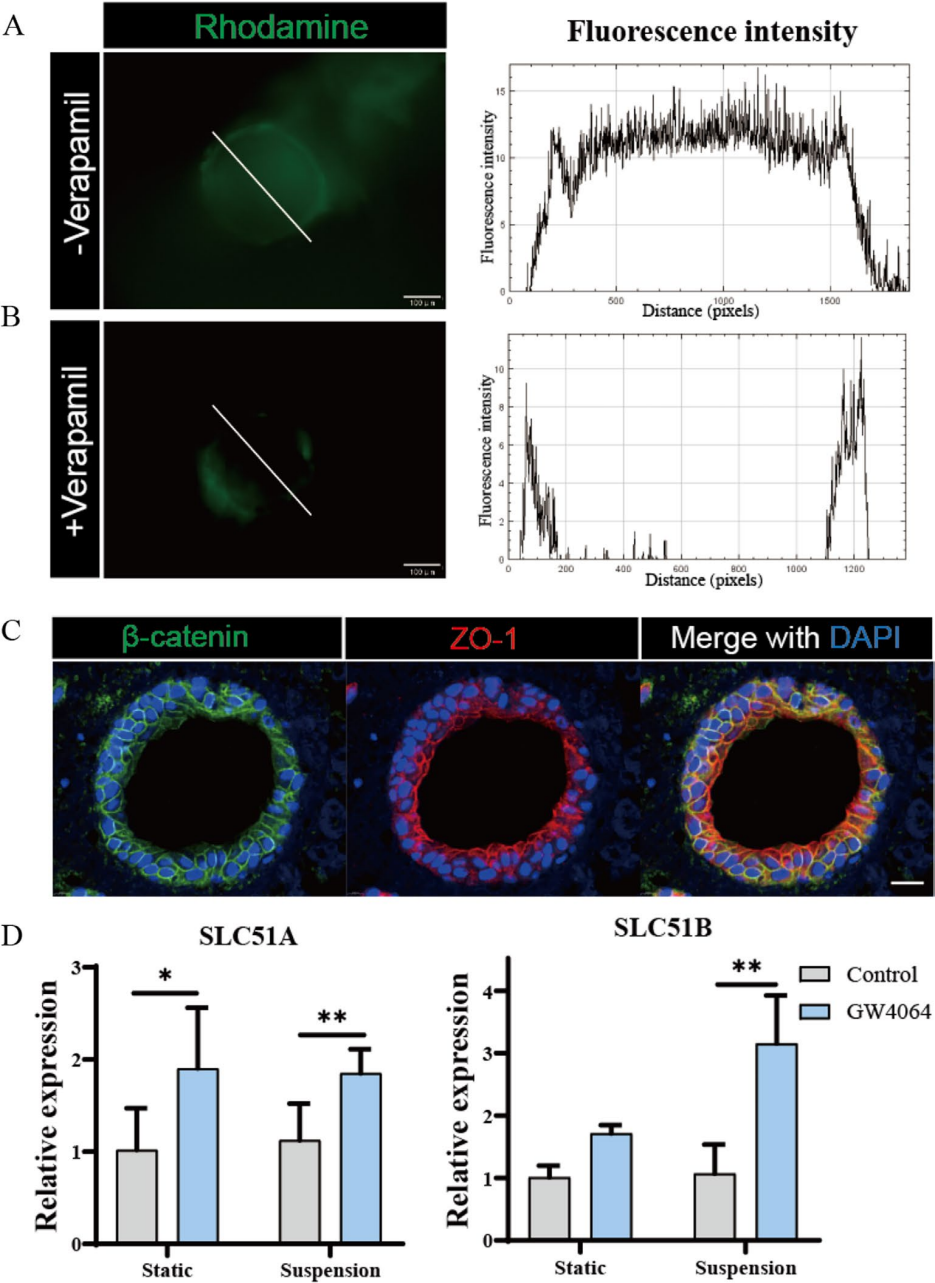
Verification of mature bile duct cell function. Display representative fluorescent micrographs of liver ductal organoids showcasing the uptake of rhodamine 123 dye under conditions of (A)verapamil treatment and (B) untreated control. Scale bar: 50 μm. Corresponding graphs quantitatively represent the fluorescence intensity measurements taken along the depicted white lines within each micrograph. The scale bars in the images are each 50 μm in length. (C) Representative images of immunofluorescence for β‐catenin and ZO‐1 in liver ductal organoids. Scale bar: 20 μm. (D) Relative expression of transporters in organoids under two culture modes in the presence and absence of GW4064.

It is necessary that functional validation is conducted on liver ductal organoids grown in suspension culture mode. Generally, the multidrug resistance protein 1 (MDR1) gene is expressed in normal liver ductal epithelial cells and is involved in the efflux of various drugs into the lumen. Thus, Rhodamine 123 was used to detect the drug efflux ability of liver duct‐like organs. After incubating the liver ductal organoids with Rhodamine 123 dye, green fluorescence was observed to accumulate in the cystic lumen of the organoids, indicating the transport role played by MDR1 (Figure 5A). In the presence of the MDR1 inhibitor verapamil, the green fluorescence was significantly reduced, and no aggregation was observed (Figure 5B). The changes in fluorescence intensity further confirmed that the liver ductal organoids actively transported the drugs through MDR1. Immunofluorescence analysis was used to localise cholangiocyte markers and assess the epithelial polarity of liver ductal organoids. As shown in Figure 5C, the organoids exhibited localised expression of ZO1 and β‐catenin, indicating that they had acquired apical‐basal polarity. The organoids under novel suspension culture primarily demonstrated apical‐basal polarity, with the apical side of the organoids facing the lumen. In the liver, a key physiological function of cholangiocytes is the regulation of bile. To evaluate the function of mature bile ductal cells in vitro, activation of the Farnesoid X receptor (FXR) was tested (Figure 5D). FXR is a bile acid‐activated transcription factor primarily distributed in the liver, ileum, and kidney. It is crucial for maintaining bile acid balance and is one of the most important nuclear receptors, promoting bile flow to prevent cholestasis. The specific organic solute transporters in bile duct cells are responsible for the excretion of bile acids (encoded by genes SCL51A and SLC51B). After treatment with the FXR activator GW4064, an increase in the expression levels of SLC51A/B was observed, consistent with nuclear receptor activation. These results indicate that mature bile ductal organoids under two different culture conditions can carry out FXR‐mediated regulation of bile acid homeostasis, which is very useful for modelling cholestasis. Conclusively, it indicates that the liver ductal organoids cultivated in suspension culture mode have good liver ductal function and can be utilised for further research.

### 3.5 Cell Viability of Dissociated Organoids

**FIGURE 6:**
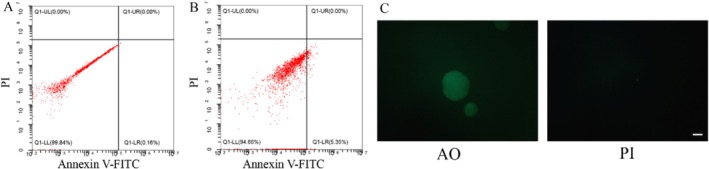
Assessment of cell viability and apoptosis in dissociated liver duct organoids. Flow cytometric analysis of apoptosis in organoids after dissociation, (A) unstained (B) double stained with Annexin V‐FITC and PI. (C) Fluorescence images of organoids after incubation with AO and PI after serial passages. Scale bar: 100 μm.

In cell transplantation, organoids must be digested into single cells before preparation for transplantation. In order to guarantee the effectiveness of transplantation cells, the quality viability of single cells from the cultured organoids dissociated by enzymatic hydrolysate was analysed. Firstly, the apoptosis of single cells after dissociation was evaluated with an Annexin V‐FITC/PI Apoptosis Detection Kit (Figure 6A,B). The typical annexin V‐FITC/PI apoptosis assay confirmed that the dissociation process hardly induced any significant apoptosis in the cells. Based on the intensity of acridine orange (green) in the organoids, it showed that liver duct organoids cultured under suspension conditions retained comparable viability after serial passages (Figure 6C). The high viability of single cells derived from cultured organoids demonstrates their suitability for cell transplantation.

### 3.6 Expansion of Organoid Culture in a Flask Bioreactor

**FIGURE 7:**
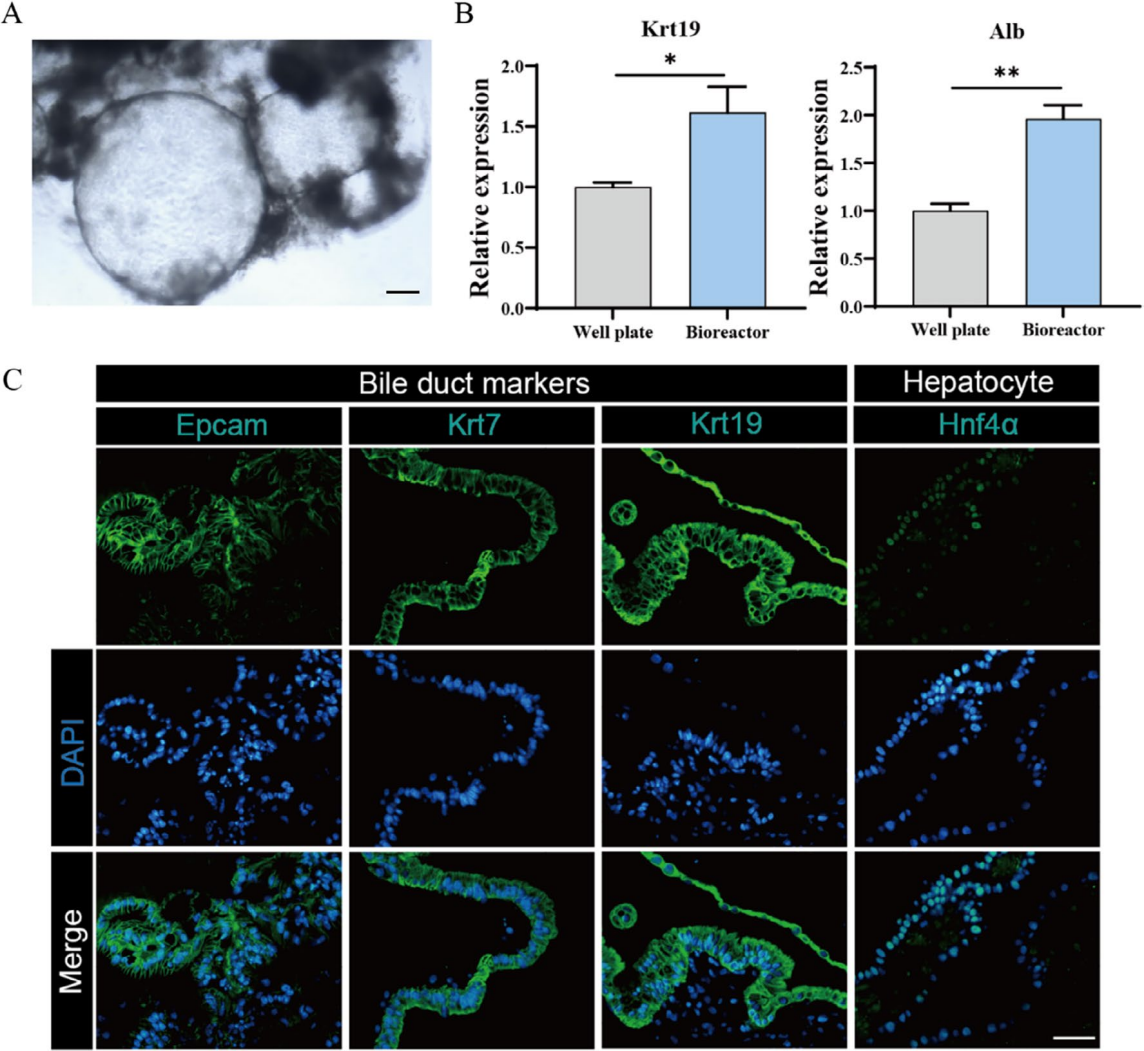
Images for liver biliary organoids and their expression of biomarker genes. (A) Images of cultured liver biliary organoids grown in spinner flask culture at day 7. Scale bar: 100 μm. (B) Expression of liver ductal genes (Krt19) and hepatocyte genes (Hnf‐4α) of organoids grown in 6‐well plates and spinner flask culture. (C) Fluorescence microscopy images of organoids grown in flask culture stained for cholangiocyte lineage markers (KRT19, KRT7, EPCAM) and hepatocyte lineage markers (HNF‐4a). Scale bar: 100 μm.

The basic cultivation method in the well plate may not be suitable for the requirement of transplanting billions of cells, necessitating a solution to meet the ever‐increasing therapeutic and pharmaceutical demand. Organoids were cultured as 3D aggregates under a dynamic suspension culture in a 50 mL spinner flask vessel with a 15 mL working volume, as previously described. The typical spherical cavity shape is revealed in Figure 7A, which proves the effectiveness of spinner flask suspension culture system. Furthermore, liver ductal organoids cultured in spinner flask exhibited obvious liver ductal characteristics as static culture condition (Figure 7B,C). From the results of immunofluorescence analyses and qPCR assays, the liver ductal organoid showed high expression of cholangiocyte lineage markers (KRT19), comparable to those in well plate cultures. These results demonstrate that the suspension culture system can further expand the culture volume to meet the large‐scale needs of liver ductal organoids.

### 3.7 Mechanosensitive Pathways Enhance the Differentiation of Liver Ductal Organoids

**FIGURE 8:**
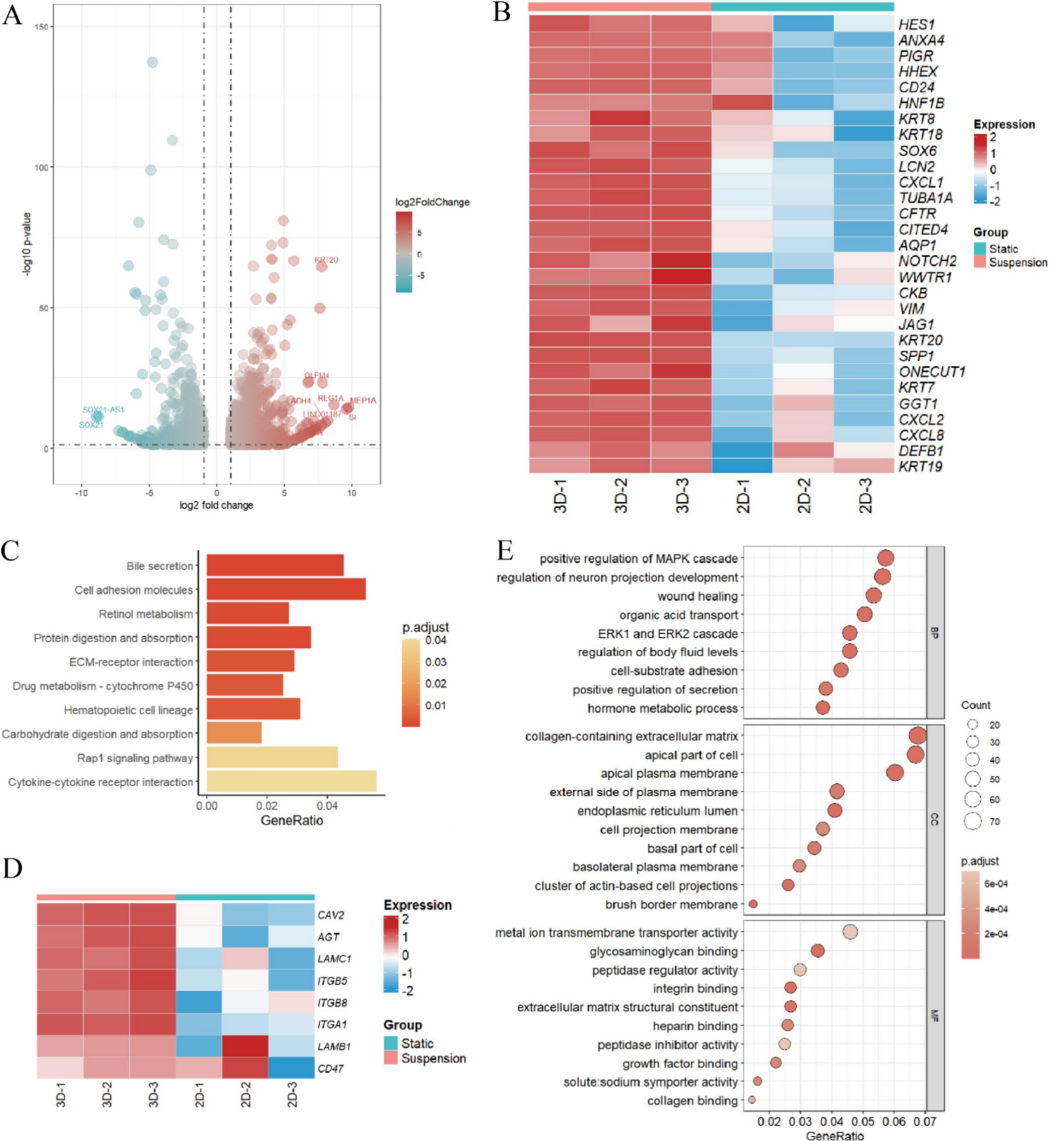
Transcriptomic profiling reveals the hepatobiliary characteristics of organoids cultured in suspension. (A) Volcano plots of DEGs that are upregulated or downregulated in Static and Suspension cultured organoids. (B) Heat map of DEGs associated with biliary characteristics. (C) KEGG analysis of DEGs between static and suspension cultured organoids. (D) The heat map of DEGs associated with cell‐matrix adhesion. (E) GO analysis of DEGs between static and suspension cultured organoids.

To gain a deeper understanding of transcriptomic changes in liver ductal organoids generated by altering the culture environment, we performed RNA sequencing on liver ductal organoids derived from three individual livers, which were cultured statically (2D) using matrigel and in suspension condition (3D). We initially conducted principal component analysis (PCA) on the variable genes under different conditions. As shown in the PCA plot and heatmap in Figures S1 and S2A, there were distinct transcriptomic profiles between the statically cultured organoids and organoids cultured in suspension, with 2059 differentially expressed genes (DEGs) (Figure 8A). Firstly, we examined the expression patterns of biliary‐specific genes (Figure 8B). Compared to static culture, suspension culture enhanced the biliary characteristics of the organoids. KRT19, KRT7, and other specific mature epithelial markers, such as PIGR, LCN2, AQP1, KRT8, and KRT18, were similarly expressed. Hepatic characteristics of the liver ductal organoids were similarly promoted (Figure S2B), with upregulation of the early hepatocyte lineage‐defining transcription factor HNF4α, which is a central regulator of hepatocyte differentiation. Of note, genes associated with cell proliferation (TOP2A, MIK67, PCNA) were highly expressed in the suspension‐cultured organoids, confirming that scaled‐up culture enhances the growth capacity of the organoids (Figure S2C). The differentiation potential of the organoids was also confirmed by the upregulation of bipotent progenitor genes, including PROX1 and HES1 (Figure S2D).

**FIGURE 9:**
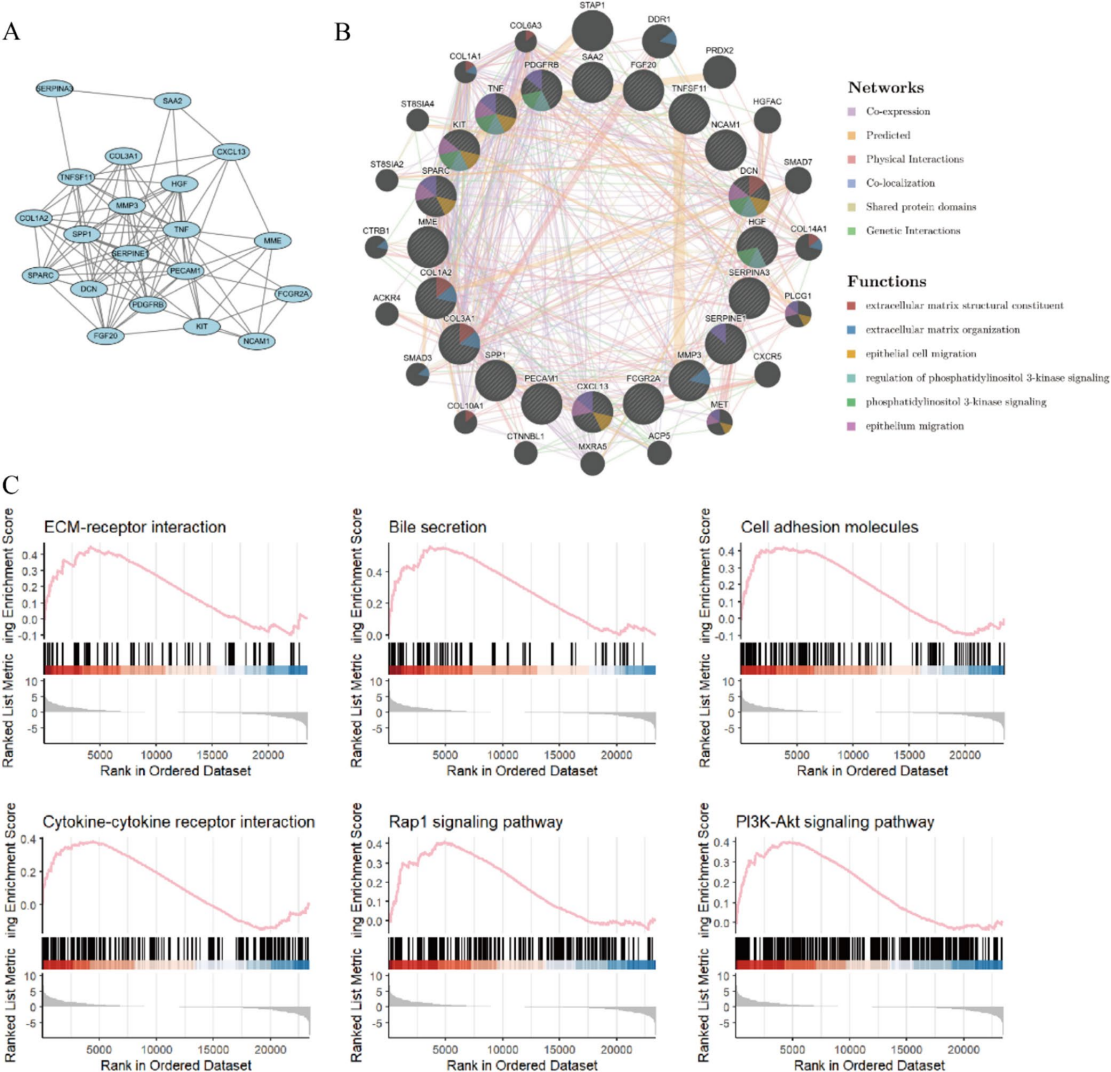
Mechanosensitive pathways enhance the differentiation of liver ductal organoids. (A) The most tightly connected gene module is derived from the MCODE plugin in Cytoscape. (B) Core DEGs and their co‐expression genes were analysed via GeneMANIA. (C) Mechanosensitive pathways enriched by GSEA.

To better understand these differences, we performed Kyoto Encyclopedia of Genes and Genomes (KEGG) analysis on the differentially expressed genes (Figure 8C). Genes that were upregulated in suspension culture were enriched in pathways related to Bile secretion and ECM‐receptor interaction, suggesting that mechanical forces in the suspension culture environment may influence gene expression through specific signalling pathways, thereby affecting the differentiation fate of cells. After searching for genes related to cell‐matrix adhesion, it was found that the majority of these genes were upregulated in suspension culture, further indicating that in the absence of ECM, external physical signals may promote cell growth (Figure 8D). Gene Ontology (GO) analysis also confirmed that genes upregulated in suspension‐cultured organoids were enriched for GO terms related to cell‐matrix adhesion and mechanotransduction (Figure 8E). MCODE plugin in Cytoscape was used for the analysis of upregulated differential genes, the results revealed the most tightly connected gene module, which included 20 significantly differentially expressed genes and 97 interacting pairs (Figure 9A). These genes are associated with extracellular matrix (ECM)‐related components, such as collagens (COL3A1, COL1A2, and COL16A1) and growth factors (HGF, FGF20). Protein co‐expression analysis indicated that these genes are involved in processes like extracellular matrix organisation, epithelial cell migration, and PI3K signalling (Figure 9B). Previous reports have indicated that the PI3K signalling pathway is regulated by mechanical forces and is one of the most important pathways for cell division, survival, and differentiation. According to Gene Set Enrichment Analysis (GSEA), suspension culture indeed significantly upregulated the expression of genes related to Cell adhesion molecules, ECM‐receptor interaction, and PI3K signalling pathways (Figure 9C). From the above results, it can be inferred that mechanical forces in suspension culture can alter the ECM‐receptor interaction pathway. The above mentioned results indicate that mechanical forces in suspension culture can modify the extracellular matrix‐receptor interaction pathway. These forces transduce external physical signals, thereby activating the PI3K signalling pathway and enhancing the differentiation and proliferation of liver ductal organoids.

### 3.8 Suspension Cultured Liver Ductal Organoids to Model Biliary Pro‐Fibrogenic Responses

**FIGURE 10:**
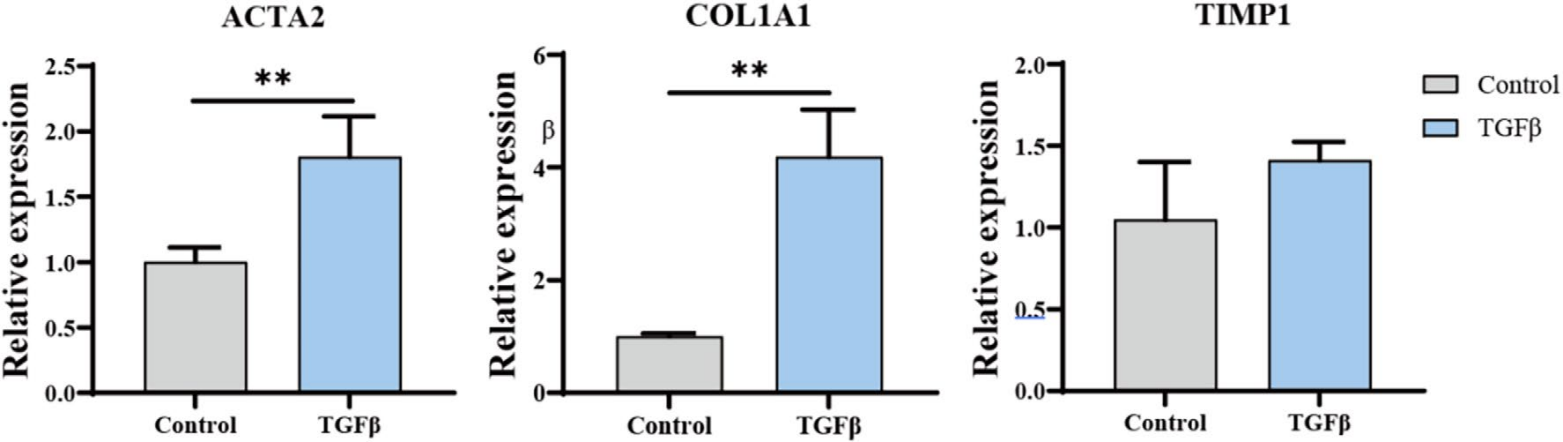
Simulation of pro‐fibrogenic responses in hepatobiliary organoids under two culture modes.

To explore the ability of these organoids to model biliary fibrosis, the impact on matrix accumulation and organ dysfunction following the activation of the TGFβ signal was detected (Figure 10). Under suspension culture conditions, organoids were cultured in the presence and absence of TGFβ (25 ng/mL) for 48 h, after which the expression of fibrosis‐related genes was assessed. The results indicated that following the activation of the TGFβ signalling pathway, the expression levels of smooth muscle actin alpha 2 (ACTA2), type I collagen alpha 1 chain (COL1A1), and tissue inhibitor of metalloproteinase 1 (TIMP1) in liver ductal organoids were upregulated. This demonstrates that suspension‐cultured liver ductal organoids can effectively model biliary pro‐fibrogenic responses, providing a potential method for efficiently constructing models of biliary pro‐fibrogenesis.

### 3.9 Suspension Cultured Liver Ductal Cell Transplantation Improves Liver Injury and Inflammation

**FIGURE 11:**
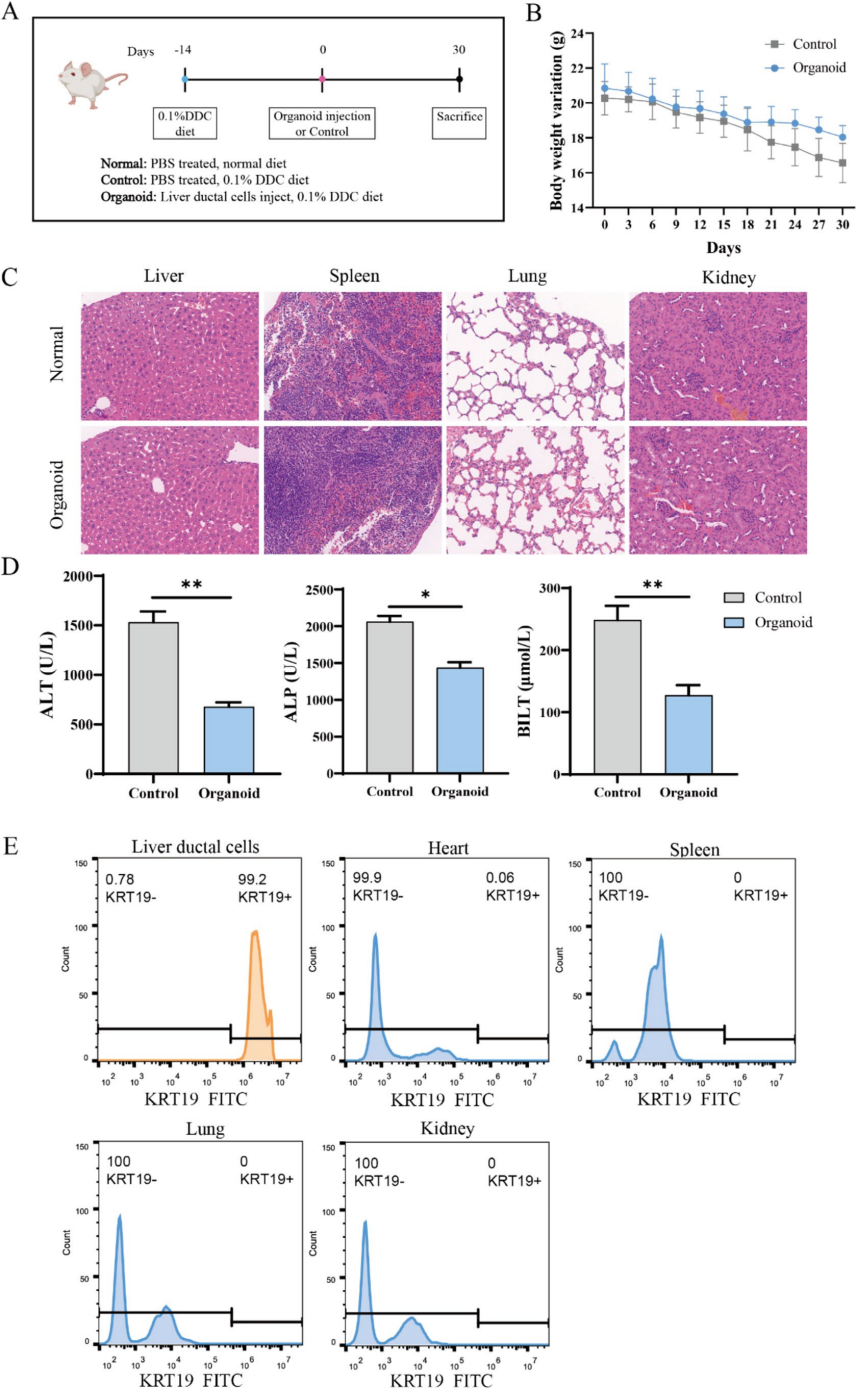
Result of liver ductal organoids transplantation into mice with liver injury. (A) Schematic diagram of mouse experiments. (B) Changes in mouse body weight during the treatment period. (C) Evaluation of biosafety of organoid transplantation. (D) Serum liver function tests (ALT, ALP, BILT). (E) Flow cytometry analysis of the proportion of KRT19+ human liver ductal cells in other organs (heart, spleen, lung and kidney) after cell isolation.

**FIGURE 12:**
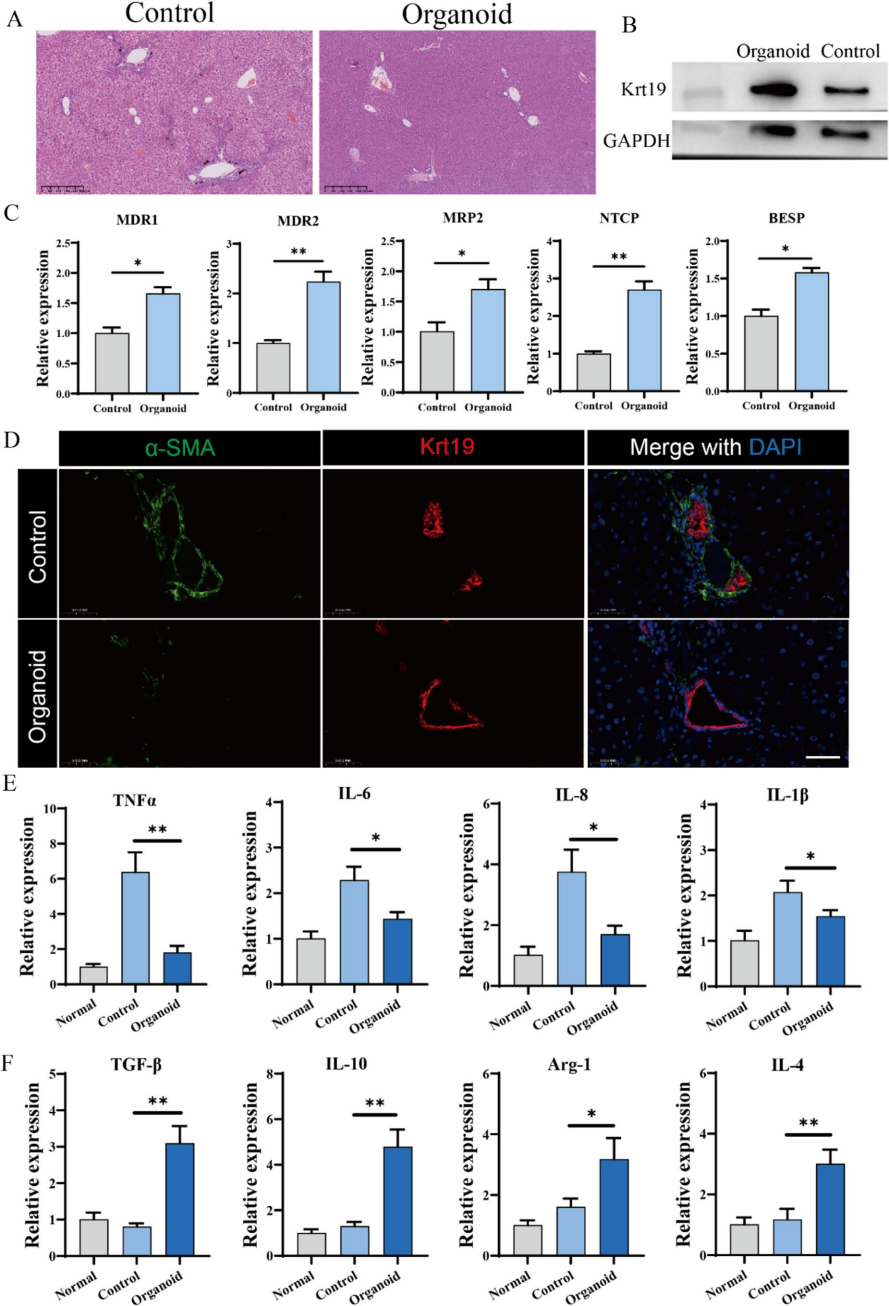
Biomarker genes and histopathological results of mice transplanted with suspension cultured liver ductal organoids. (A) Representative images of HE staining in liver tissue. (B) Western‐blot results of krt19 in liver tissue. (C) Relative expression of bile acid transport genes in liver tissue (MDR1, MDR2, MRP2, NTCP, BESP). (D) Representative images of immunofluorescence for the key fibrotic marker (α‐SMA) and the bile duct marker (Krt19) in liver tissue. Scale bar: 50 μm. (E) Relative expression of pro‐inflammatory genes in liver tissue (IL‐8, IL‐6, TNFα, IL‐1β). (F) Relative expression of anti‐inflammatory genes in liver tissue (IL‐4, Arg1, IL‐10, TGFβ).

To verify whether the novel suspension cultured liver ductal organoids have the potential to repair liver damage, suspension cultured liver ductal organoids were used for transplantation (Figure 11A). Compared with healthy mice, the transplantation had no effect on the main organs, indicating the in vivo safety of liver ductal cell transplantation (Figure 11C). After treating liver injury with liver ductal cell transplantation, it was found that the transplantation alleviated the weight loss of mice (Figure 11B). Compared with mice with liver injury, the serum liver function indicators ALT, ALP, BLIT significantly decreased after transplantation, indicating that cell transplantation improved liver injury (Figure 11D). Importantly, after in situ cell transplantation, flow cytometry was performed to detect the proportion of KRT19+ human liver ductal cells in other organs (heart, spleen, lung, kidney) after cell dissociation. The results showed that no human liver ductal cells engrafted to other organs after transplantation, which proved the safety of in situ cell transplantation (Figure 11E). Histopathological examination of liver sections revealed that compared with mice with liver injury, mice treated with cell transplantation had less cholestasis, cholangiocyte proliferation with inflammatory infiltration, and milder hepatocyte necrosis (Figure 12A). Western‐blot results of liver tissue also showed an increasing trend of the cholangiocyte marker krt19 after cell transplantation (Figure 12B). Furthermore, the relative expression of bile acid transport genes (MDR1, MDR2, MRP2, NTCP, BESP) in liver tissue was examined, and the results showed an increase of bile acid transport genes after cell transplantation, which may be due to the repair of cholangiocyte injury and the alleviation of cholestasis (Figure 12C). Cell transplantation also alleviated biliary injury as shown in Figure 12D. Biliary fibrosis was obviously reduced after treatment, and the characteristics of the bile ducts were restored, demonstrating that hepatobiliary organoids have good engraftment effects. Meanwhile, the relative expression of pro‐inflammatory genes (IL‐8, IL‐6, TNFα, IL‐1β) and anti‐inflammatory genes (IL‐4, Arg1, IL‐10, TGFβ) in the transplanted group was detected, and it was found that liver ductal cell transplantation reduced pro‐inflammatory genes while enhancing anti‐inflammatory genes (Figure 12E,F). Overall, the findings from animal studies indicate that hepatobiliary organoids derived from suspension culture exhibit favourable engraftment following transplantation and are capable of significantly mitigating liver fibrosis and inflammation. The capability to generate liver ductal organoids at large scale presents promising avenues for their use in regenerative medicine and as an alternative to traditional tissue transplants.

## 4 Discussion

In this work, a highly efficient and matrigel‐free suspension cultivation method for generating self‐renewing 3D liver ductal organoids has been developed to achieve considerable and high‐quality organoids. This was followed by the determination of cell viability, cell lineage of generated liver ductal organoids, and western‐blot verification for liver ductal biomarkers. The expansion of liver ductal organoid culture was further amplified in a 50 mL flask bioreactor. Furthermore, the transplantation of suspension cultured liver ductal organoids into mice was carried out, indicating that it could improve liver injury and inflammation. Thus, we provide a new perspective on organoid culture, highlighting the functionality and stemness characteristics of liver ductal organoids, particularly in large‐scale cultivation without matrigel.

(1) Novel matrigel‐free suspension culture enhances the generation of high‐quality human liver ductal organoids.

Organoids have been reported for fundamental mechanistic studies on organ development, medical regeneration and repair in human tissues [10, 51, 52]. They can also be applied in diagnostics, disease modelling, drug discovery and personalised medicine [53, 54, 55]. Currently, the advantages of organoids in the field of regenerative medicine are limited by their culturing efficiency because organoids are mainly derived from either pluripotent or tissue‐resident stem cells or progenitor or differentiated cells from healthy or diseased tissues [13, 56, 57, 58, 59]. For the application of organoids in regenerative medicine, ensuring a substantial cell yield is essential to meet the demands of transplantation. Till now, numerous organoid engineering strategies that support organoid culture and growth, proliferation, differentiation and maturation have been reported (Table 1). Generally, there are two culture modes for organoid generation. One is a static culture that involves embedding organoid cells within extracellular matrix (ECM) to form a 3D (static) scaffold that supports the development of cells into organoids. Another one is a dynamic culture, namely suspension culture that directly cultivates organoids in liquid medium and is non adherent dependent. The former method for organoid culture relies on extracellular matrices (ECM) for cell growth, proliferation, and self‐assembly to form self‐renewing 3D organoids with the structure and function of the corresponding in vivo tissue. For example, a wide variety of organoids have been successfully cultured, encompassing multiple human tissues and organs, such as intestinal organoids [23], brain organoids [62], liver organoids [11], kidney organoids [63] and lung organoids [64]. However, there are some drawbacks in static organoid culture: (1) the primary components are sourced from natural matrices of animal origin. The presence of animal‐derived components may compromise the organoids' fidelity in simulating human organs [31]. (2) Traditional static culture techniques have not yet achieved full standardisation, and scaling up cell production requires increased reagents and labor costs, thereby limiting the clinical application of organoids. For instance, although previous studies have provided methods for the culture of hepatocyte organoids, they still rely on matrigel and have not achieved standardised production of organoids, which hinders further application of organoids [10, 51].

**TABLE 1:** Some examples of enhanced organoid culture by static and suspension modes.

Organoid	Culture modes	Outcomes	References
Hepatocyte organoid	Static cultivation requires matrigel and complex operation	Long‐term expansion of functional mouse and human hepatocytes as 3D organoids.	Hu et al. [10]
Liver and pancreas organoid	Static cultivation requires matrigel and complex operation	Establishment of self‐renewing human and mouse adult liver and pancreas 3D organoids	Broutier et al. [51]
Gastrointestinal organoids	Static cultivation with novel extracellular matrix hydrogels	Tissue extracellular matrix hydrogels as alternatives to Matrigel for culturing gastrointestinal organoids	Kim et al. [31]
Lung organoid	Static cultivation with novel soluble ECM	Soluble ECM was developed to promote organotypic formation in lung alveolar model	Valdoz et al. [44]
Liver biliary organoid	Suspension culture requires a mixture of 10% ECM and culture medium	Facile suspension culture protocol of the liver biliary organoids was developed	Chen et al. [43]
Colorectal cancer organoid	Suspension culture requires a mixture of 5% ECM and culture medium	A suspension technique for efficient large‐scale cancer organoid culturing and perturbation screens	Price et al. [33]
Gastrointestinal organoid	Suspension culture, but the establishment of organoids requires ECM	Controlling the polarity of human gastrointestinal organoids by suspension culture	Co et al. [60]
Gastrointestinal organoid	Suspension culture with polymer‐hydrogel substrate and microengineered cell culture device	High‐throughput automated organoid culture via stem‐cell aggregation in microcavity arrays	Brandenberg et al. [61]
Bile ductal organoid	Suspension culture, no need for ECM throughout the process.	A novel matrigel‐free suspension culture enhances the generation of high‐quality human liver ductal organoids. Growth rate and size exceeding twice that of the static state.	In this study

In order to overcome the shortcomings of static organoid culture, suspension culture presents a novel approach to large‐scale organoid cultivation, promising to overcome the limitations of ECM and enhance the efficiency of the culture process. While previous reports have explored suspension culture methods, they have not fully mitigated the influence of matrigels [33, 43, 65], and the culture process still necessitates complex operations. The culture systems previously described are mainly confined to well plates [46, 66, 67], which are inadequate for large‐scale production needs. In this work, we introduce a matrigel‐free culture method that significantly improves the production efficiency and functionality of organoids. Firstly, high‐quality organoids exhibit a higher growth rate compared to static culture to generate liver ductal organoids. (1) Suspension culture enhances the growth rate of hepatobiliary organoids, with an average diameter 2.6 times that of static culture (Figure 1). (2) Suspension culture enhances the expression of hepatobiliary markers, indicating improved functionality (Figures 3, 4, 5). Therefore, a matrigel‐free culture reported here can generate robust organoids. Secondly, this study preliminarily expanded the culture system of organoids and validated the large‐scale suspension culture of organoids using flask bioreactor. In spinner flask culture, organoids maintain a superior phenotype compared to static conditions. The cell yield from suspension culture is 2.3 times higher than that from static culture (Figure 2C). Consequently, this study demonstrates excellent scalability for organoid culture. Thirdly, the controllable process of suspension organoid culture can be amplified at larger scales. Bioreactors allow for the optimization of operational parameters such as agitation speed, aeration rate, temperature, and pH during the scale‐up process to meet the demands of large‐scale cultivation. In contrast, static culture lacks precise control over the cultivation process, making scale‐up challenging [68, 69]. The suspension culture within the bioreactor system enables the surveillance of cellular cultivation with a multiplicity of parameters, thereby facilitating the development of systems for the suspension expansion and differentiation of organoids. Such an approach holds the promise of yielding a substantial quantity of organoids in a single batch, suitable for cellular therapeutics or high‐throughput drug screening assays. In short, the novel suspension culture method significantly enhances the generation and quality of liver ductal organoids. From the transcriptomic profiling analysis, it is evident that mechanical forces provide dynamic biochemical cues and mechanical stimuli, driving cellular mechanotransduction through mechanosensitive pathways, such as the YAP signalling pathway [39, 50, 70]. These pathways play a significant role in cell differentiation and proliferation, ultimately leading to accelerated cellular differentiation and proliferation. Additionally, the mass transfer microenvironment in suspension culture is notably improved, likely due to the enhanced mass transfer and gas exchange provided by the suspension culture system. This, in turn, promotes better growth and functional maintenance of organoids.

Previous studies have demonstrated that clinical hepatobiliary organoid transplantation requires approximately 1 × 10^8^ cells per dose [22]. Computational modelling suggests that a single 200 mL batch of suspension culture could meet this demand, whereas traditional adherent methods would necessitate hundreds of multi‐well plates and extended time investments. While our current matrigel‐free suspension culture system operates at a 15 mL scale—a volume not yet optimised for clinical translation—scaling poses inherent challenges due to altered mixing flows and dispersion heterogeneities, which may compromise the reproducibility of organoid quality observed at smaller scales. Given the high costs associated with organoid biomanufacturing, future investigations must prioritise the following multidisciplinary strategies: (1) Agitation system optimization: Refinement of impeller design and rotation speed to achieve homogeneous cell distribution, mitigate aggregate formation, and preserve critical cell–cell and cell‐matrix interactions—parameters essential for maintaining organoid structural integrity and functional maturation. (2) Particle image velocimetry (PIV) analysis: Quantitative mapping of velocity fields and turbulence characteristics within the suspension culture system using fluorescent tracer particles. This approach will elucidate flow patterns and shear stress distributions acting on organoids, enabling data‐driven optimization of hydrodynamic conditions to balance mixing efficiency and mechanical protection. (3) Computational fluid dynamics (CFD) modelling: Development of multiphase CFD simulations to predict flow regimes, mass transfer rates, and shear stress profiles across scales (15 to 200 mL). By correlating simulated parameters with experimental outcomes (organoid viability, marker expression), this framework will guide the design of bioreactor geometries and operating conditions that minimise scale‐up variability. Collectively, these engineering strategies will establish a predictive scaling pathway, ensuring consistent production of high‐quality organoids while reducing manufacturing costs compared to matrigel‐dependent platforms.

(2) The suspension cultured liver ductal organoids show remarkable efficacy in model construction and mouse transplantation.

Compared to traditional two‐dimensional cell line models, organoids have unique advantages in drug screening and precision medicine. High‐throughput drug screening will inevitably require a large number of cells to meet the demand in the future [71]. In this study, we tested the pro‐fibrotic phenotype induced by TGFβ signalling. Our research results show that after TGFβ stimulation based on gene expression, organoids exhibited a pro‐fibrotic phenotype, proving that the scaled preparation of organoids may improve the efficiency of drug screening.

In a murine model of biliary disorders, which are hallmarked by inflammation, fibrosis, and biliary stenosis, we have assessed the regenerative capabilities of liver ductal organoids cultured in suspension. The transplantation of these organoids resulted in a significant reduction in inflammation and fibrosis, as evidenced by decreased transaminase levels, indicating an amelioration of biochemical liver function. Moreover, the transplantation of suspension cultured organoids facilitated the restoration of biliary architecture. The observed mitigation of overall hepatic inflammation and fibrosis underscores the broader regenerative potential of suspension‐cultured organoids beyond the intrahepatic biliary system, implicating a possible paracrine effect of the transplanted cells. Future investigations should delineate the differential signalling pathways between suspension and static cultures to identify the factors that concurrently enhance the proliferation of suspension‐cultured organoids and preserve their functional integrity.

## 5 Conclusion

Traditional static culture methods impede the clinical application of organoids in regenerative medicine. This study presents a novel suspension culture method for liver ductal organoids without matrigel and preliminarily explores the potential applications of suspension cultured liver ductal organoids. Transcriptomic profiling and biological experiments confirmed that the suspension culture system is more conducive to promoting the growth and maintenance of organoids through mechanical forces and enhancing hepatobiliary characteristics via the PI3K signalling pathway. The scale‐up culture system in flask bioreactors significantly enhances the efficiency of organoid batch culture. More importantly, the efficacy of transplantation with suspension cultured organoids has been successfully demonstrated through animal experiments, providing a research foundation for the potential application of alternative tissue and organ substitutes. This safe and rapid strategy may revolutionise the culture mode of organoids to meet the growing demands in various biomedical and pharmaceutical applications.

## Author Contributions

**Senyi Gong:** methodology, validation, investigation, data curation, Visualisation, writing – original draft, writing – review and editing. **Kangxin He:** methodology, formal analysis, validation, visualisation. **Yu Liu, Xingyu Luo, Kamran Ashraf and Jinzhao He:** formal analysis, validation, visualisation. **Weifeng Li, Lihua Yang, Touseef Ur Rehman, Mingwei Shen and Qinbiao Yan:** formal analysis, validation, visualisation. **Ali Mohsin, Shusen Zheng, Zhe Yang and Meijin Guo:** supervision, writing – review and editing, funding, project administration.

## Ethics Statement

Liver tissues were collected during liver surgery at Shulan (Hangzhou) Hospital in Hangzhou, China. The use of human tissues for research purposes has been approved by the Medical Ethics Committee of Shulan (Hangzhou) Hospital (Ethical Approval Number: KY2024020). All procedures of the animal experiments have been approved by the institutional animal care and use committee of Shanghai Jiaotong University (Ethical Approval Number: A2024136). All studies were performed in accordance with the ethical standards as laid down in the 1964 Declaration of Helsinki.

## Conflicts of Interest

The authors declare no conflicts of interest.

## Supporting information


**Data S1.** Supporting Information.

## Data Availability

The data that support the findings of this study are available on request from the corresponding author. The data are not publicly available due to privacy or ethical restrictions.

## References

[1] R. Pais , A. S. Barritt , Y. Calmus , et al., “NAFLD and Liver Transplantation: Current Burden and Expected Challenges,” Journal of Hepatology 65, no. 6 (2016): 1245–1257, 10.1016/j.jhep.2016.07.033. 27486010 PMC5326676

[2] W. A. Dar , E. Sullivan , J. S. Bynon , H. Eltzschig , and C. Ju , “Ischaemia Reperfusion Injury in Liver Transplantation: Cellular and Molecular Mechanisms,” Liver International 39, no. 5 (2019): 788–801. 30843314 10.1111/liv.14091 PMC6483869

[3] F. Durand , J. Levitsky , F. Cauchy , H. Gilgenkrantz , O. Soubrane , and C. Francoz , “Age and Liver Transplantation,” Journal of Hepatology 70, no. 4 (2019): 745–758. 30576701 10.1016/j.jhep.2018.12.009

[4] H. Mergental , M. T. P. R. Perera , R. W. Laing , et al., “Transplantation of Declined Liver Allografts Following Normothermic Ex‐Situ Evaluation,” American Journal of Transplantation 16, no. 11 (2016): 3235–3245, 10.1111/ajt.13875. 27192971

[5] A. Sanchez‐Fueyo , G. Whitehouse , N. Grageda , et al., “Applicability, Safety, and Biological Activity of Regulatory T Cell Therapy in Liver Transplantation,” American Journal of Transplantation 20, no. 4 (2020): 1125–1136. 31715056 10.1111/ajt.15700 PMC7154724

[6] G. Mazza , K. Rombouts , A. R. Hall , et al., “Decellularized Human Liver as a Natural 3D‐Scaffold for Liver Bioengineering and Transplantation,” Scientific Reports 7, no. 5 (2015): 13079. 10.1038/srep13079 PMC4528226 26248878

[7] O. B. van Leeuwen , S. B. Bodewes , V. A. Lantinga , et al., “Sequential Hypothermic and Normothermic Machine Perfusion Enables Safe Transplantation of High‐Risk Donor Livers,” American Journal of Transplantation 22, no. 6 (2022): 1658–1670. 35286759 10.1111/ajt.17022 PMC9325426

[8] L. Campana and J. P. Iredale , “Regression of Liver Fibrosis,” Seminars in Liver Disease 37, no. 1 (2017): 1–10. 28201843 10.1055/s-0036-1597816

[9] C. C. Bell , D. F. G. Hendriks , S. M. L. Moro , et al., “Characterization of Primary Human Hepatocyte Spheroids as a Model System for Drug‐Induced Liver Injury, Liver Function and Disease,” Scientific Reports 4, no. 6 (2016): 25187. 10.1038/srep25187 PMC4855186 27143246

[10] H. Hu , H. Gehart , B. Artegiani , et al., “Long‐Term Expansion of Functional Mouse and Human Hepatocytes as 3D Organoids,” Cell 175, no. 6 (2018): 1591–1606. 30500538 10.1016/j.cell.2018.11.013

[11] N. Prior , P. Inacio , and M. Huch , “Liver Organoids: From Basic Research to Therapeutic Applications,” Gut 68, no. 12 (2019): 2228–2237. 31300517 10.1136/gutjnl-2019-319256 PMC6872443

[12] G. Rossi , A. Manfrin , and M. P. Lutolf , “Progress and Potential in Organoid Research,” Nature Reviews. Genetics 19, no. 11 (2018): 671–687. 10.1038/s41576-018-0051-9 30228295

[13] R. Cruz‐Acuna , M. Quiros , A. E. Farkas , et al., “Synthetic Hydrogels for Human Intestinal Organoid Generation and Colonic Wound Repair,” Nature Cell Biology 19, no. 11 (2017): 1326–1335. 29058719 10.1038/ncb3632 PMC5664213

[14] L. Chung , D. R. Maestas, Jr. , F. Housseau , and J. H. Elisseeff , “Key Players in the Immune Response to Biomaterial Scaffolds for Regenerative Medicine,” Advanced Drug Delivery Reviews 114 (2017): 184–192. 28712923 10.1016/j.addr.2017.07.006

[15] X.‐L. Fan , Y. Zhang , X. Li , and Q.‐L. Fu , “Mechanisms Underlying the Protective Effects of Mesenchymal Stem Cell‐Based Therapy,” Cellular and Molecular Life Sciences 77, no. 14 (2020): 2771–2794. 31965214 10.1007/s00018-020-03454-6 PMC7223321

[16] Z. Julier , A. J. Park , P. S. Briquez , and M. M. Martino , “Promoting Tissue Regeneration by Modulating the Immune System,” Acta Biomaterialia 53 (2017): 13–28. 28119112 10.1016/j.actbio.2017.01.056

[17] F. Cofano , M. Boido , M. Monticelli , et al., “Mesenchymal Stem Cells for Spinal Cord Injury: Current Options, Limitations, and Future of Cell Therapy,” International Journal of Molecular Sciences 20, no. 11 (2019): 2698, 10.3390/ijms20112698. 31159345 PMC6600381

[18] J. Etulain , “Platelets in Wound Healing and Regenerative Medicine,” Platelets 29, no. 6 (2018): 556–568. 29442539 10.1080/09537104.2018.1430357

[19] G. Itakura , S. Kawabata , M. Ando , et al., “Fail‐Safe System Against Potential Tumorigenicity After Transplantation of iPSC Derivatives,” Stem Cell Reports 8, no. 3 (2017): 673–684. 28262544 10.1016/j.stemcr.2017.02.003 PMC5355810

[20] Y. Nakamura , S. Miyagawa , S. Yoshida , et al., “Natural Killer Cells Impede the Engraftment of Cardiomyocytes Derived From Induced Pluripotent Stem Cells in Syngeneic Mouse Model,” Scientific Reports 9, no. 1 (2019): 10840. 31346220 10.1038/s41598-019-47134-3 PMC6658523

[21] J. Shiota , L. C. Samuelson , and N. Razumilava , “Hepatobiliary Organoids and Their Applications for Studies of Liver Health and Disease: Are We There Yet?,” Hepatology 74, no. 4 (2021): 2251–2263. 33638203 10.1002/hep.31772 PMC9067600

[22] F. Sampaziotis , D. Muraro , and O. C. Tysoe , “Cholangiocyte Organoids Can Repair Bile Ducts After Transplantation in the Human Liver,” Science 371, no. 6531 (2021): 839–846. 33602855 10.1126/science.aaz6964 PMC7610478

[23] N. Glorevski , N. Sachs , A. Manfrin , et al., “Designer Matrices for Intestinal Stem Cell and Organoid Culture,” Nature 539, no. 7630 (2016): 560–564. 27851739 10.1038/nature20168

[24] M. T. Kozlowski , C. J. Crook , and H. T. Ku , “Towards Organoid Culture Without Matrigel,” Communications Biology 4, no. 1 (2021): 1387. 34893703 10.1038/s42003-021-02910-8 PMC8664924

[25] M. A. G. Barbosa , C. P. R. Xavier , R. F. Pereira , V. Petrikaite , and M. H. Vasconcelos , “3D Cell Culture Models as Recapitulators of the Tumor Microenvironment for the Screening of Anti‐Cancer Drugs,” Cancers 14, no. 1 (2022): 190. 10.3390/cancers14010190 PMC8749977 35008353

[26] C. Jubelin , J. Munoz‐Garcia , L. Griscom , et al., “Three‐Dimensional In Vitro Culture Models in Oncology Research,” Cell and Bioscience 12, no. 1 (2022): 155. 36089610 10.1186/s13578-022-00887-3 PMC9465969

[27] W. Kim , Y. Gwon , S. Park , H. Kim , and J. Kim , “Therapeutic Strategies of Three‐Dimensional Stem Cell Spheroids and Organoids for Tissue Repair and Regeneration,” Bioactive Materials 19 (2023): 50–74. 35441116 10.1016/j.bioactmat.2022.03.039 PMC8987319

[28] E. A. Aisenbrey and W. L. Murphy , “Synthetic Alternatives to Matrigel,” Nature Reviews Materials 5, no. 7 (2020): 539–551. 10.1038/s41578-020-0199-8 PMC7500703 32953138

[29] N. Bock , F. Forouz , L. Hipwood , et al., “GelMA, Click‐Chemistry Gelatin and Bioprinted Polyethylene Glycol‐Based Hydrogels as 3D Ex Vivo Drug Testing Platforms for Patient‐Derived Breast Cancer Organoids,” Pharmaceutics 15, no. 1 (2023): 261, 10.3390/pharmaceutics15010261. 36678890 PMC9867511

[30] N. Carpentier , S. C. Ye , M. D. Delemarre , et al., “Gelatin‐Based Hybrid Hydrogels as Matrices for Organoid Culture,” Biomacromolecules 25, no. 2 (2024): 590–604. 38174962 10.1021/acs.biomac.2c01496

[31] S. Kim , S. Min , Y. S. Choi , et al., “Tissue Extracellular Matrix Hydrogels as Alternatives to Matrigel for Culturing Gastrointestinal Organoids,” Nature Communications 13, no. 1 (2022): 1692. 10.1038/s41467-022-29279-4 PMC8967832 35354790

[32] T. Lokai , B. Albin , K. Qubbaj , A. P. Tiwari , P. Adhikari , and I. H. Yang , “A Review on Current Brain Organoid Technologies From a Biomedical Engineering Perspective,” Experimental Neurology 367 (2023): 114461. 37295544 10.1016/j.expneurol.2023.114461

[33] S. Price , S. Bhosle , E. Goncalves , et al., “A Suspension Technique for Efficient Large‐Scale Cancer Organoid Culturing and Perturbation Screens,” Scientific Reports 12, no. 1 (2022): 5571. 35368031 10.1038/s41598-022-09508-y PMC8976852

[34] O. Chaudhuri , J. Cooper‐White , P. A. Janmey , D. J. Mooney , and V. B. Shenoy , “Effects of Extracellular Matrix Viscoelasticity on Cellular Behaviour,” Nature 584, no. 7822 (2020): 535–546. 32848221 10.1038/s41586-020-2612-2 PMC7676152

[35] F. Meng , C. Shen , L. Yang , et al., “Mechanical Stretching Boosts Expansion and Regeneration of Intestinal Organoids Through Fueling Stem Cell Self‐Renewal,” Cell Regeneration 11, no. 1 (2022): 39, 10.1186/s13619-022-00137-4. 36319799 PMC9626719

[36] M. Wang , X. Zhou , S. Zhou , et al., “Mechanical Force Drives the Initial Mesenchymal‐ Epithelial Interaction During Skin Organoid Development,” Theranostics 13, no. 9 (2023): 2930–2945. 37284452 10.7150/thno.83217 PMC10240816

[37] N. Kobayashi , S. Togo , T. Matsuzaki , et al., “Stiffness Distribution Analysis in Indentation Depth Direction Reveals Clear Mechanical Features of Cells and Organoids by Using AFM,” Applied Physics Express 13, no. 9 (2020): 7001–7004.

[38] T. Matsuzaki , Y. Shimokawa , H. Koike , et al., “Mechanical Guidance of Self‐Condensation Patterns of Differentiating Progeny,” Iscience 25, no. 10 (2022): 105109. 36317160 10.1016/j.isci.2022.105109 PMC9617469

[39] A. Elosegui‐Artola , I. Andreu , A. E. M. Beedle , et al., “Force Triggers YAP Nuclear Entry by Regulating Transport Across Nuclear Pores,” Cell 171, no. 6 (2017): 1397–1410. 29107331 10.1016/j.cell.2017.10.008

[40] T. Iskratsch , H. Wolfenson , and M. P. Sheetz , “Appreciating Force and Shape—The Rise of Mechanotransduction in Cell Biology,” Nature Reviews. Molecular Cell Biology 15, no. 12 (2014): 825–833. 25355507 10.1038/nrm3903 PMC9339222

[41] K. H. Vining and D. J. Mooney , “Mechanical Forces Direct Stem Cell Behaviour in Development and Regeneration,” Nature Reviews. Molecular Cell Biology 18, no. 12 (2017): 728–742. 29115301 10.1038/nrm.2017.108 PMC5803560

[42] N. Aizarani , A. Saviano , N. Sagar , et al., “A Human Liver Cell Atlas Reveals Heterogeneity and Epithelial Progenitors,” Nature 572, no. 7768 (2019): 199–204. 31292543 10.1038/s41586-019-1373-2 PMC6687507

[43] J. Chen , J. Zhang , L. Yang , and B. Zhao , “Facile Suspension Culture Protocol of the Liver Biliary Organoids,” Bio‐Design and Manufacturing 6, no. 1 (2023): 74–81.

[44] J. C. Valdoz , N. A. Franks , C. G. Cribbs , et al., “Soluble ECM Promotes Organotypic Formation in Lung Alveolar Model,” Biomaterials 283 (2022): 121464. 35306229 10.1016/j.biomaterials.2022.121464 PMC9359416

[45] Y.‐C. Lu , D.‐J. Fu , D. An , et al., “Scalable Production and Cryostorage of Organoids Using Core‐Shell Decoupled Hydrogel Capsules,” Advanced Biosystems 1, no. 12 (2017): 1700165, 10.1002/adbi.201700165. 29607405 PMC5870136

[46] E. D. Wrenn , B. M. Moore , E. Greenwood , M. McBirney , and K. J. Cheung , “Optimal, Large‐Scale Propagation of Mouse Mammary Tumor Organoids,” Journal of Mammary Gland Biology and Neoplasia 25, no. 4 (2020): 337–350. 33106923 10.1007/s10911-020-09464-1 PMC7587543

[47] T. Zhou , B. Gao , Y. Fan , et al., “Piezo1/2 Mediate Mechanotransduction Essential for Bone Formation Through Concerted Activation of NFAT‐YAP1‐Ss‐Satenin,” eLife 9 (2020): e52779. 32186512 10.7554/eLife.52779 PMC7112954

[48] H.‐Q. Xu , J.‐C. Liu , Z.‐Y. Zhang , and C.‐X. Xu , “A Review on Cell Damage, Viability, and Functionality During 3D Bioprinting,” Military Medical Research 9, no. 1 (2022): 70. 36522661 10.1186/s40779-022-00429-5 PMC9756521

[49] X. Di , X. Gao , L. Peng , et al., “Cellular Mechanotransduction in Health and Diseases: From Molecular Mechanism to Therapeutic Targets,” Signal Transduction and Targeted Therapy 8, no. 1 (2023): 282. 37518181 10.1038/s41392-023-01501-9 PMC10387486

[50] B. C. Low , C. Q. Pan , G. V. Shivashankar , A. Bershadsky , M. Sudol , and M. Sheetz , “YAP/TAZ as Mechanosensors and Mechanotransducers in Regulating Organ Size and Tumor Growth,” FEBS Letters 588, no. 16 (2014): 2663–2670. 24747426 10.1016/j.febslet.2014.04.012

[51] L. Broutier , A. Andersson‐Rolf , C. J. Hindley , et al., “Culture and Establishment of Self‐Renewing Human and Mouse Adult Liver and Pancreas 3D Organoids and Their Genetic Manipulation,” Nature Protocols 11, no. 9 (2016): 1724–1743. 27560176 10.1038/nprot.2016.097

[52] Z. Zhao , X. Chen , A. M. Dowbaj , et al., “Organoids,” Nature Reviews Methods Primers 2, no. 1 (2022): 94, 10.1038/s43586-022-00174-y. PMC10270325 37325195

[53] H. Xu , X. Lyu , M. Yi , W. Zhao , Y. Song , and K. Wu , “Organoid Technology and Applications in Cancer Research,” Journal of Hematology and Oncology 11, no. 1 (2018): 116. 30219074 10.1186/s13045-018-0662-9 PMC6139148

[54] S. Maharjan , C. Ma , B. Singh , et al., “Advanced 3D Imaging and Organoid Bioprinting for Biomedical Research and Therapeutic Applications,” Advanced Drug Delivery Reviews 208 (2024): 115237, 10.1016/j.addr.2024.115237. 38447931 PMC11031334

[55] S. Gunti , A. T. K. Hoke , K. P. Vu , and N. R. London , “Organoid and Spheroid Tumor Models: Techniques and Applications,” Cancers 13, no. 4 (2021): 874. 33669619 10.3390/cancers13040874 PMC7922036

[56] J. A. Brassard , M. Nikolaev , T. Huebscher , M. Hofer , and M. P. Lutolf , “Recapitulating Macro‐Scale Tissue Self‐Organization Through Organoid Bioprinting,” Nature Materials 20, no. 1 (2021): 22–29. 32958879 10.1038/s41563-020-00803-5

[57] X.‐Y. Tang , S. Wu , D. Wang , et al., “Human Organoids in Basic Research and Clinical Applications,” Signal Transduction and Targeted Therapy 7, no. 1 (2022): 168. 35610212 10.1038/s41392-022-01024-9 PMC9127490

[58] S. J. Mun , J.‐S. Ryu , M.‐O. Lee , et al., “Generation of Expandable Human Pluripotent Stem Cell‐Derived Hepatocyte‐Like Liver Organoids,” Journal of Hepatology 71, no. 5 (2019): 970–985. 31299272 10.1016/j.jhep.2019.06.030

[59] D. Garnier , R. Li , F. Delbos , et al., “Expansion of Human Primary Hepatocytes In Vitro Through Their Amplification as Liver Progenitors in a 3D Organoid System,” Scientific Reports 8, no. 1 (2018): 8222. 29844473 10.1038/s41598-018-26584-1 PMC5974235

[60] J. Y. Co , M. Margalef‐Català , D. M. Monack , and M. R. Amieva , “Controlling the Polarity of Human Gastrointestinal Organoids to Investigate Epithelial Biology and Infectious Diseases,” Nature Protocols 16, no. 11 (2021): 5171–5192. 34663962 10.1038/s41596-021-00607-0 PMC8841224

[61] N. Brandenberg , S. Hoehnel , F. Kuttler , et al., “High‐Throughput Automated Organoid Culture via Stem‐Cell Aggregation in Microcavity Arrays,” Nature Biomedical Engineering 4, no. 9 (2020): 863–874. 10.1038/s41551-020-0565-2 32514094

[62] A. A. Mansour , J. T. Goncalves , C. W. Bloyd , et al., “An In Vivo Model of Functional and Vascularized Human Brain Organoids,” Nature Biotechnology 36, no. 5 (2018): 432–441. 10.1038/nbt.4127 PMC6331203 29658944

[63] R. Morizane , A. Q. Lam , B. S. Freedman , S. Kishi , M. T. Valerius , and J. V. Bonventre , “Nephron Organoids Derived From Human Pluripotent Stem Cells Model Kidney Development and Injury,” Nature Biotechnology 33, no. 11 (2015): 1193–1200. 10.1038/nbt.3392 PMC4747858 26458176

[64] Y.‐W. Chen , S. X. Huang , A. L. , de de Rodrigues Toste Carvalho , et al., “A Three‐Dimensional Model of Human Lung Development and Disease From Pluripotent Stem Cells,” Nature Cell Biology 19, no. 5 (2017): 542–549, 10.1038/ncb3510. 28436965 PMC5777163

[65] J. Brugge , K.‐C. Chang , F. Silvestri , et al., “Breast Organoid Suspension Cultures Maintain Long‐Term Estrogen Receptor Expression and Responsiveness,” Research Square 3 (2024): 4463390. 10.1038/s41523-024-00714-7 PMC11659324 39702422

[66] Y. Takahashi , S. Sato , Y. Kurashima , et al., “A Refined Culture System for Human Induced Pluripotent Stem Cell‐Derived Intestinal Epithelial Organoids,” Stem Cell Reports 10, no. 1 (2018): 314–328. 29233552 10.1016/j.stemcr.2017.11.004 PMC5768885

[67] P. Kakni , R. Hueber , K. Knoops , et al., “Intestinal Organoid Culture in Polymer Film‐Based Microwell Arrays,” Advanced Biosystems 4, no. 10 (2020): e2000126. 32734713 10.1002/adbi.202000126

[68] V. Sander , A. Przepiorski , N. A. Hukriede , and A. J. Davidson , “Large‐Scale Production of Kidney Organoids From Human Pluripotent Stem Cells,” in Kidney Research: Experimental Protocols, vol. 2664 (Springer US, 2023), 69–83. 10.1007/978-1-0716-3179-9_6 37423983

[69] J. P. P. Licata , K. H. H. Schwab , Y.‐e. Har‐el , J. A. A. Gerstenhaber , and P. I. I. Lelkes , “Bioreactor Technologies for Enhanced Organoid Culture,” International Journal of Molecular Sciences 24, no. 14 (2023): 11427. 37511186 10.3390/ijms241411427 PMC10380004

[70] I. Dasgupta and D. McCollum , “Control of Cellular Responses to Mechanical Cues Through YAP/TAZ Regulation,” Journal of Biological Chemistry 294, no. 46 (2019): 17693–17706. 31594864 10.1074/jbc.REV119.007963 PMC6873206

[71] H. Ryoo , H. Kimmel , E. Rondo , and G. H. Underhill , “Advances in High Throughput Cell Culture Technologies for Therapeutic Screening and Biological Discovery Applications,” Bioengineering and Translational Medicine 9, no. 3 (2024): e10627. 38818120 10.1002/btm2.10627 PMC11135158

