# Supplementary material for: Scalable Matrigel‐Free Suspension Culture for Generating High‐Quality Human Liver Ductal Organoids
Source: Cell Prolif. 2025 Apr 1;58(9):e70033. doi: 10.1111/cpr.70033 (PMC12414634; doi:10.1111/cpr.70033)
Supplement: Supplementary file 1 — Data S1. Supporting Information. [file CPR-58-e70033-s001.docx]

**Supplementary Information**

**Research article**

**Scalable Matrigel-Free Suspension Culture for Generating High-Quality Human Liver Ductal Organoids**

**Table S1. Table of QPCR primers used.**

| gene name |  | Primer sequence (5´-3´) |
| --- | --- | --- |
| KRT19 | Forward | CTTCCGAACCAAGTTTGAGAC |
| KRT19 | Reverse | AGCGTACTGATTTCCTCCTC |
| ALB | Forward | GCACAGAATCCTTGGTGAACAG |
| ALB | Reverse | ATGGAAGGTGAATGTTTCAGCA |
| KRT7 | Forward | GAAGCATGGGGACGACCT |
| KRT7 | Reverse | CACGAGCATCCTTGAGCG |
| PROM1 | Forward | GGACAAGGCGTTCACAGATC |
| PROM1 | Reverse | TGGTCTCCTTGATCGCTGTT |
| CYP3A4 | Forward | TTCAGCAAGAAGAACAAGGACAA |
| CYP3A4 | Reverse | GGTTGAAGAAGTCCTCCTAAGC |
| SOX9 | Forward | ATGAAGATGACCGACGAGCA |
| SOX9 | Reverse | CAGTCGTAGCCTTTGAGCAC |
| GAPDH | Forward | GTCAGTGGTGGACCTGACCT |
| GAPDH | Reverse | TGCTGTAGCCAAATTCGTTG |
| SLC51A | Forward | TTGTTCGCCTCCCTATTCC |
| SLC51A | Reverse | TTGTGGTCTTTCCTTCGGT |
| SLC51B | Forward | TGTGGTGGTCATTATAAGCATGG |
| SLC51B | Reverse | TCTTAGGTTGTTTAGGCTGTTGTG |
| ACTA2 | Forward | GTGTTGCCCCTGAAGAGCAT |
| ACTA2 | Reverse | GCTGGGACATTGAAAGTCTCA |
| COL1A1 | Forward | TCCAACGAGATCGAGATCC |
| COL1A1 | Reverse | AAGCCGAATTCCTGGTCT |
| TIMP1 | Forward | CTTCTGGCATCCTGTTGTTG |
| TIMP1 | Reverse | GGTATAAGGTGGTCTGGTTG |
| NTCP | Forward | CAAACCTCAGAAGGACCAAACA |
| NTCP | Reverse | GTAGGAGGATTATTCCCGTTGTG |
| BSEP | Forward | TCTGACTCAGTGATTCTTCGCA |
| BSEP | Reverse | GTGTAGAGTGAAGTCCTCCTTAGC |
| MDR1 | Forward | ACACTTGGCCCCAAACATAGA |
| MDR1 | Reverse | GTCAATGCTTGGCTCGTTATCA |
| MDR2 | Forward | CGGCGACTTTGAACTAGGCA |
| MDR2 | Reverse | CAGAGTATCGGAACAGTGTCAAC |
| MRP2 | Forward | ACGTTTAGTTGGTATGACAGCAC |
| MRP2 | Reverse | TGCTTCTTGGTCAATCCGTGT |
| IL-6 | Forward | GGTGATATTCGAGACCATTTACTG |
| IL-6 | Reverse | GCCAACAGTAGCCTTCACCCAT |
| IL-8 | Forward | GGTGATATTCGAGACCATTTACTG |
| IL-8 | Reverse | GCCAACAGTAGCCTTCACCCAT |
| TNFα | Forward | CTCTTCTGCCTGCTGCACTTTG |
| TNFα | Reverse | ATGGGCTACAGGCTTGTCACTC |
| IL-1β | Forward | TGGACCTTCCAGGATGAGGACA |
| IL-1β | Reverse | GTTCA TCTCGGAGCCTGTAGTG |
| Arg1 | Forward | CATTGGCTTGCGAGACGTAGAC |
| Arg1 | Reverse | GCTGAAGGTCTCTTCCATCACC |
| IL-10 | Forward | CGGGAAGACAATAACTGCACCC |
| IL-10 | Reverse | CGGTTAGCAGTATGTTGTCCAGC |
| TGFβ | Forward | TGATACGCCTGAGTGGCTGTCT |
| TGFβ | Reverse | CACAAGAGCAGTGAGCGCTGA |
| IL-4 | Forward | ATCATCGGCATTTTGAACGAGGTC |
| IL-4 | Reverse | ACCTTGGAAGCCCTACAGACGA |


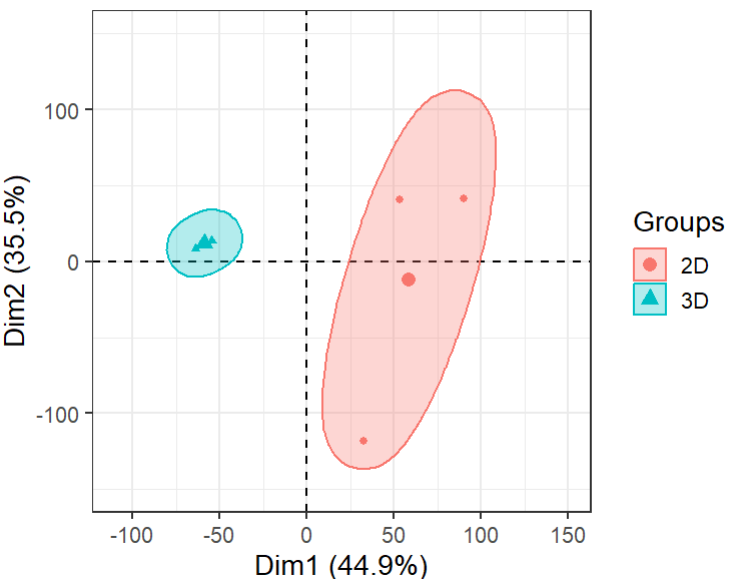


**Fig. S1.** Principal component analysis of organoids cultured under static (2D) and dynamic conditions (3D).


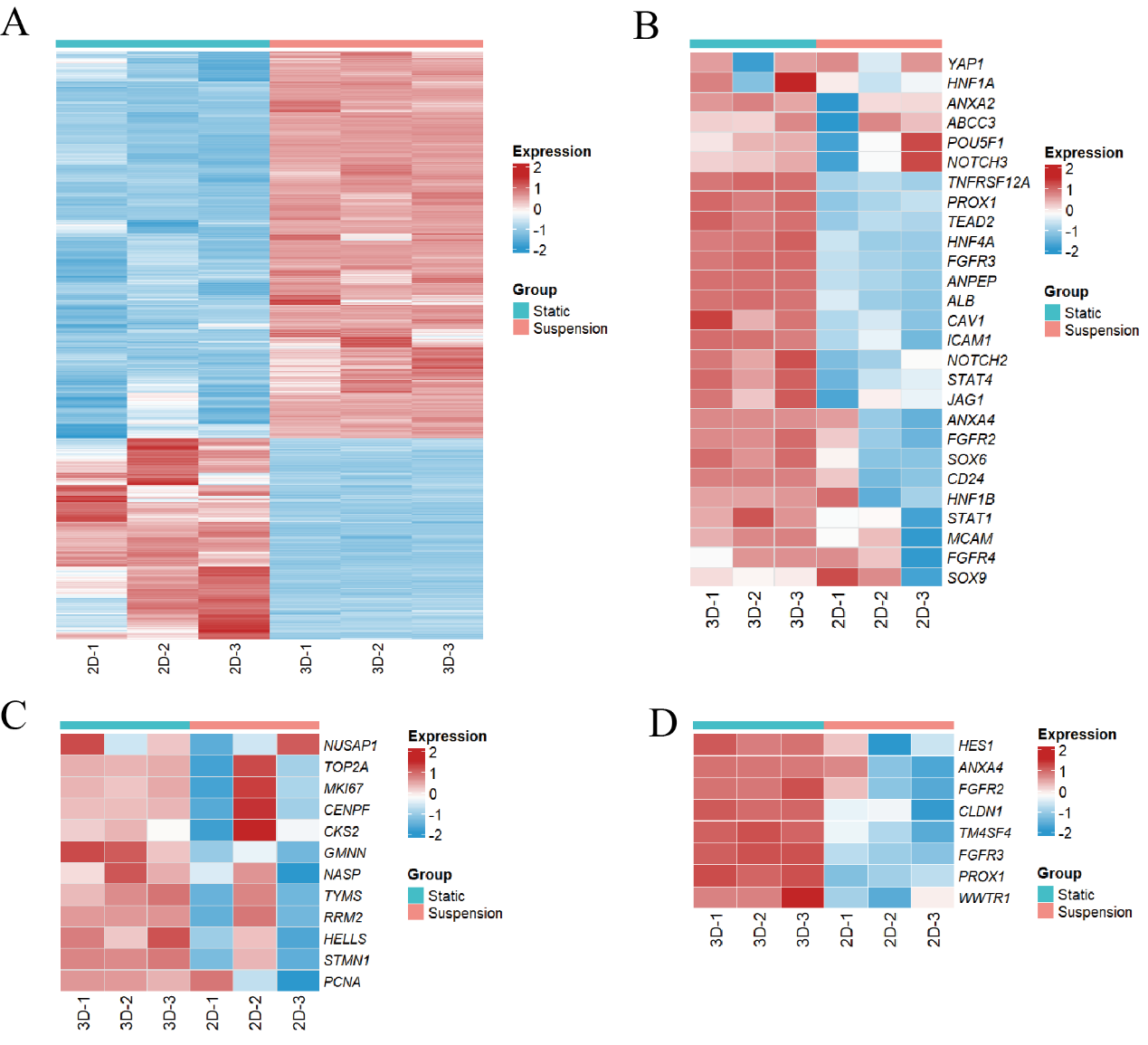


**Fig. S2.** (A) Heatmap of DEGs in organoids generated from static and suspension conditions. (B) The heat map of DEGs associated with Hepatic characteristics. (C) The heat map of DEGs associated with cell proliferation. (D) The heat map of DEGs associated with bipotent progenitor genes.
